# Efficient feature ranked hybrid framework for android Iot malware detection

**DOI:** 10.1038/s41598-026-35238-6

**Published:** 2026-01-27

**Authors:** Nahla Hafez Saeed, Alyaa A. Hamza, Mohamed A. Sobh, Ayman M. Bahaa-Eldin

**Affiliations:** 1 Computer Engineering & Systems Department, Faculty of Engineering, Ain Shams University, Cairo, Egypt; 2 Elsewedy University of Technology, Cairo, Egypt

**Keywords:** Artificial intelligence (AI), Random forest classification, IoT security, Hybrid analysis, Android malware detection, Computational biology and bioinformatics, Engineering, Mathematics and computing

## Abstract

Android-based IoT devices are still exposed to increasing sophistication in malware; therefore, detecting this malware using lightweight and accurate approaches is very important. This paper presents a hybrid malware detection framework, incorporating static and dynamic analysis with a dual feature-ranking mechanism based on Information Gain and Gini Index, for selecting the most relevant features. The framework uses a Random Forest classifier optimized via systematic hyperparameter tuning and is evaluated on four benchmark datasets: Drebin, CCCS-CIC-AndMal-2020, TUANDROMD, and CIMD-2024. It showed very consistent performance across all four datasets, yielding accuracy in the range of 99.03% to 100% with corresponding F1-scores in the range of 0.98 to 1.00. On the contrary, the highly imbalanced nature of the CIMD-2024 dataset essentially requires imbalance-handling strategies to effectively detect both majority and minority classes. Experiments on cross-validation confirm the model’s stability and generalizability, while the interpretability analysis pinpoints the most influential behavioral and static features that drive such classification. The results ensured that the proposed approach provided an efficient, interpretable, and resource-friendly solution for malware detection within Android-IoT environments.

## Introduction

 With the global rise and interconnectivity of smart devices, IoT has grown tremendously in all aspects. These devices enable autonomous sensing, monitoring, control, and data sharing in various domains, including smart grid, transportation, agriculture, cities, health, and public safety. Most of these IoT devices operate on Android because of its open-source nature, flexibility, and easy integration with heterogeneous hardware. Accordingly, remote control and sensing system applications interested in IoT have become quite popular with gadgets like smart TVs, cars, refrigerators, and wearable devices. Within this ecosystem, smartphones improve services with environmental sensing and home security supervision, acting as a convenient graphical user interface with NFC, RFID, WLAN, and Bluetooth^1,2^.

The most recent statistics report around 3 billion active Android devices as Internet of Things devices, emphasizing the ubiquity of the platform^3^. However, its popularity is also synonymous with attracting the attention of cyber threats. Attackers exploit vulnerabilities in Android to compromise security, loot personal information, and control IoT networks. This threat is further sharpened by the absence of built-in security provisions and reliance of its users on third-party app sources^4,5^. Studies showed that most of the Android IoT devices do not implement basic antivirus protection and have thus remained unprotected against targeted as well as opportunistic malware campaigns^6,7^. Consequently, previously developed techniques that can be applied to the malware mitigation techniques include sandboxing^8^, signature-based detection^9^, behavioral monitoring^10^, and permission-based access control^11,12^.

Recent Android and IoT malware incidents indicate the importance of enhancing detection systems. For example, the TrickBot malware family has compromised more than 250 million email accounts and over 140,000 devices worldwide from major companies such as Microsoft, PayPal, and Amazon, causing serious operational disruption. Moreover, new 2024–2025 threats including the GoldPickaxe biometric-stealing Trojan, Mirai-based IoT botnets launching large-scale DDoS attacks, and evolving Android spyware families such as SpyNote, each show just how fast contemporary malware is evolving. The above incidents signify the requirement for lightweight, accurate, generalizable hybrid detection frameworks that can handle emerging real-world threats^13^. Also, Android malware relies increasingly on obfuscation, API-call manipulation, and adversarial evasion techniques to successfully evade both static and deep-learning-based detectors by more than 50% in some systems. These evolving attack strategies underscore the growing urgency for robust, generalizable, and lightweight detection frameworks resistant to real-world evasion attempts^14^. Recent research in 2025 unveiled large-scale exploitation of Android devices using AI-powered dynamic malware; attackers had used on-device machine learning APIs to disguise malicious behaviors during execution. Such an incident revealed how malware could change its behavior at runtime, thus evading traditional signature-based defenses and exposing a critical vulnerability in modern Android security^15^.

This work focuses on providing a hybrid framework for malware detection in Android-based IoT environments. The proposed model employs an RF classifier trained exclusively on both static and dynamic features from hybrid analysis according to which the static features are the permission sets, the API usage, and manifest metadata, while the dynamic features include runtime behaviors and traffic patterns. RF is chosen because it has proven to be the best in handling high-dimensional feature spaces, is still resistant to noise, and is efficient even in the low-resource environment; thus, deployment on IoT edge devices would be suitable^16^. The framework incorporates a structured preprocessing pipeline and systematic hyperparameter optimization to ensure maximum performance, scalability, and robustness across diverse malware samples.

In this scenario, the paper exploits a dual feature-ranking scheme that applies Information Gain and Gini Index. The hybridism of the two approaches lowers the feature space dimensionality but retains considerable discriminations peak thereby increasing model interpretability and inference efficiency^17,18^. Experimental validation is through the application of four well-adopted Android malware datasets: Drebin TUANDROMD,, CCCS-CIC-AndMal-2020 CIMD-2024. The proposed system achieves 99.03% and 100% accuracy on these datasets, respectively, proving competitive with a slight extra computation.

Nevertheless, in Android malware detection, CNNs, RNNs, and hybrid DL-based strategies^19,20^ today considered weighed down by heavy computation and thereby precluded from being used in a constrained IoT environment. Static or signature-only methods further fail in following the track of evolving obfuscation and new malware behavior^9,21^. Hence there is an increasing demand for lightweight models whose working mechanisms are interpretable, capable of accurate prediction, and offer practical deployment in mission-critical areas, including healthcare, transportation, and smart infrastructure^22,23^.

In contrast to much more computationally intensive CNNs and hybrid deep-learning models, Random Forest (RF) gives a lightweight and transparent means of malware detection for the Android-IoT domain. RF naturally performs well with high-dimensional hybrid features such as permissions, API calls, and intents, without requiring large balanced datasets to train or common pre-processing with vice versa considerations. Featured-ranking from RF produces interpretability on the feature level, allowing analysts identify the strongest indicators of malware behavior, which adds to contextually appropriate interpretations especially for trusted IoT environments^24^.

Additionally, RF is adaptive to noisy and imbalanced datasets, mitigates overfitting with ensemble averaging, and is capable of realizing inference in real-time for IoT compatible usecases in constrained environments. RF resilience is further enhanced by the ensemble nature of the approach and generally less susceptible to adversarial attacks compared to deep-learning approaches, and offers an overall improvement to soundness in IoT applications over deep-learning approaches^25^.

Some of the recently developed frameworks, such as MLDroid^26^, DeepAMD^27^, MAPAS^28^, and Chybridroid^29–31^ have achieved dynamic combinations of static and behavioral cues. However, many of these models compromise trust-level accuracy for the sake of complexity or transparency in feature contribution—this is an important factor in trust-centric IoT scenarios. Few models do tests on standardized datasets such as (Drebin, TUANDROMD CCCS-CIC-AndMal-2020 and CIMD-2024). Most of the previous ones discussed hybrid learning^26–29^ but seldom addressed the issues of deployment feasibility, interpretability, or real-time performance evaluations on standardized datasets. The research addresses these issues head-on via a lightweight explanatory framed, rigorously benchmarked approach. Our research positions itself squarely in addressing those aspects by building a transparent dual feature ranked RF model, comprehensively evaluated with realistic assumptions.

In contrast to prior hybrid or deep learning-based detection systems, our approach uniquely balances accuracy, interpretability, and computational efficiency. To bridge these gaps, this paper makes the following key contributions:

- A lightweight hybrid detection framework is proposed employing Random Forest, with a static and dynamic analysis scheme to improve the efficiency of malware detection in the Android-based IoT structures.
- Design a dual ranking of features by Information Gain and Gini Index for Optimizing Dimensionality and enhance model interpretability on it.
- The proposed model is evaluated against the three benchmark datasets: Drebin and TUANDROMD, CCCS-CIC-AndMal-2020 and CIMD-2024 accomplishing exceptional accuracy.
- Establish real-time applicability because of very low computational overhead with additional explainability through feature importance visualizations compared to deep learning.

The rest of the paper is organized in the following manner: Section “Related work” does a survey of the state-of-the-art static, dynamic, and hybrid malware detection methods. Section “Proposed AndroidIoT security systems framework:” elaborates on the design and workflow of the novel hybrid scheme. Section “Experimental evaluation” sets forth the dataset, prep, and measures for evaluation. Experimental results, performance analyses, and visualization of feature importance are all presented in Section “Discussion”. While deployment constraints and the challenges, tackled in Section “Conclusion and future work”, are examined. Finally, the paper ends in Section Conclusion and future work, mentioning future directions under consideration.

## Related work

It provides nearly all aspects of android malware detection under different analysis paradigms- static, dynamic and also hybrid- improving the precision and robustness of threat classification in environments with low resources like IoT. In this section, an overview of strengths and weaknesses of these paradigms, with special emphasis on recent frameworks and gaps that our method hopes to fill.

### Static analysis techniques

Static analysis examines applications without executing them, obtaining evidence based on structure from permissions, intents, or API calls. Static analysis is lightweight and intuitive; hence, it is useful in resource-constrained environments such as IoT-based devices. The major limiting factor is its susceptibility to obfuscation and encryption techniques that hide malicious logic and hamper any pattern recognition. In the arena of Android malware detection, static approaches increasingly face robustness issues related to the ascertainment of zero-day and polymorphic threats. Since signature-based techniques assume the presence of a well-defined fingerprint of known malware, new variants evade their detection. Permission-based detection, which assesses applications by their declared permissions^32^, is also poorly defended against advanced evasion techniques. Static analysis may then form a rapid and reasonable engine for malware detection, but it stands to be improved-upon, often through dynamic or hybrid approaches-for improved detection of modern threats^33^. A notable framework for static detection is Drebin^9^, which has pioneered the use of a bag-of-words declaration incorporating permissions, intent filters, and restricted API calls to create feature vectors. Several follow-up studies^11,34,35^ focused on secure learning methods like Sec-SVM for improving resilience against adversarial attacks. MLDroid^26^ took the static analysis further. The comparison of various features such as detection methods, classification techniques, and performance on static android malware detection techniques is explained in Table 1.

**Table 1:** A comparison of related work based on static analysis techniques.

Ref	Features	Accuracy	ML technique	Contribution	Recommendations
^11^	Permissions, API calls, intents	90%	SVM	The paper proposes a secure-learning paradigm to enhance the robustness of machine learning models, specifically targeting Android malware detection against well-crafted evasion attacks.	The authors suggest extending the secure-learning approach to other malware detection tasks and integrating dynamic analysis, recommend applying secure-learning paradigms to other security tasks and continuing research on adversary-aware machine learning techniques.
^34^	Permissions	91.7%	Random forest	Introduces a secure-learning paradigm, specifically the Sec-SVM algorithm, to enhance the robustness of Android malware detection against evasion attacks	The authors recommend applying the secure-learning approach to other security tasks and advocate for further research on integrating static and dynamic analysis methods to improve system security.
^35^	Permissions, API calls, intents	93.7%	SVM	The development of Sec-SVM, a secure-learning algorithm that enhances resilience against evasion attacks.	Applying secure-learning paradigms to other security tasks and continuing research on adversary-aware machine learning techniques, extending the secure-learning approach to other malware detection tasks and integrating dynamic analysis
^26^	Permissions, API calls, intents	98.8%	SVM, MLP	The paper presents MLDroid, a web-based Android malware detection framework that effectively uses permissions and API calls to distinguish between benign and malicious apps.	Exploring additional machine learning models and feature selection methods to further enhance malware detection capabilities, the application of MLDroid in real-world scenarios for detecting both known and unknown malware families efficiently.
^19^	Opcode, API features, and permission	98%	DNN	Android malware detection framework that utilizes multiple static features and a multimodal deep learning approach to improve detection accuracy	Exploring integrating dynamic features with the existing static feature-based framework to enhance detection capabilities, using the proposed multimodal deep learning approach as it effectively improves the accuracy of Android malware detection​
^27^	API calls, permissions, intents	93.4%	Deep ANN	The paper introduces DeepAMD, a novel approach for detecting and identifying Android malware, which outperforms existing methods by enhancing detection accuracy on both static and dynamic layers​	Developing an online service to allow users to check whether an application is benign or malicious before downloading it​, using DeepAMP for effective and accurate detection and identification of Android malware, as it shows superior performance compared to other existing techniques
^36^	Permissions	97%	MLP, KNN and Random forest	The research evaluates 49 malware families using extensive datasets and demonstrates the effectiveness of machine learning classifiers in mobile malware detection.	The authors suggest further exploration of dynamic analysis techniques and broader datasets to improve detection accuracy, integrating anomaly-based intrusion detection systems with machine learning.
^37^	Permissions	98.6%	J48	A framework based on SIGPID was developed to extract 22 specific permissions.	The authors suggest that the DJDL model could involve exploring different loss functions and batch composition schemes, applying the model to other domains, incorporating temporal information, and evaluating it on more challenging datasets.
^28^	API calls	91.27%	CNN	MAPAS provides a practical Android malware detection system that efficiently identifies malware using a lightweight classifier based on API call graphs.	The authors plan to address limitations related to obfuscated API calls that cannot be analyzed by Flowdroid, utilizing MAPAS for effective and efficient malware detection on resource-limited devices
^38^	Permissions	94.9%	Random forest, J48	Introduced a comprehensive comparison of feature selection methods and classification algorithms for Android malware detection.	Suggested exploring additional feature extraction techniques and real-time malware detection methods using Random Forest and J48 Decision Tree for permission-based malware detection.
^39^	Permissions	99.3%	KNN, NB, SMO ,MLP, RF, LR	The study demonstrates the effectiveness of using Principal Component Analysis (PCA) and Linear Discriminant Analysis (LDA) for dimensionality reduction, significantly improving the runtime and performance of machine learning classifiers for Android malware detection	The authors suggest further exploration of other dimensionality reduction techniques and machine learning algorithms to enhance the detection accuracy and efficiency of the proposed system using dimension reduction techniques like PCA and LDA to improve the performance and speed of machine learning models in Android malware detection
^21^	API calls	93.4%	Signature matching	Development of the Androguard tool for effective reversing and decomplication of Android applications.	Exploration of further enhancements in malware detection and obfuscation evaluation techniques, encouragement for further research in improving static analysis and reverse engineering tools for Android applications.
^40^	Permissions, API call	99.3%	Random Forest, MLP, AdaBoost, SVM, and Decision Tree	Introducing an approach for detecting Android malware, demonstrating the use of LSTM for rapid-sequence-snapshot analysis of API and system calls​	the study suggests the need to develop long-term sustainable security solutions by better understanding malware and good ware behavior over time.
^41^	API calls	98.26%	CNN	Proposed the novel use of an adjacency matrix approach for Android malware detection with CNN	Extend the use of this embedding method to different types of binaries​, the technique of embedding Android applications for neural network training is effective in malware classification
^20^	Permissions, API calls, and filtered intents	98.96%	GRU	Proposed a scalable, CUDA-empowered GRU-based malware detection technique for multi-class malware detection	Improving the speed efficiency of the proposed GRU model​, the efficient and timely detection of malware can significantly help in preventing attacks​
^42^	Permission	98.14%	RF, EL	The paper provides a comprehensive survey of features for detecting Android malicious applications, highlighting key issues and future directions	Focus on exploring effective features and improving detection methods against evolving malicious behaviors, emphasize the development of hybrid analysis techniques to enhance detection accuracy and efficiency
^43^	APK permissions, opcodes	99%	Deep Neural Decision Forest (DNDF)	This paper proposes a resource-aware deep learning framework to optimize computation and memory-related metrics in relation to Android malware detection. It further improves the detection accuracy while reducing the runtime overhead, and hence, such feasibility is accomplished on resource-constrained devices.	A deep learning-centric Android malware identification system that prioritizes the optimization of computational and memory resources has been developed. This project advocate for the continuation of their work through the investigation of hardware-aware optimizations like model pruning and quantization, along with the evaluation of the method on bigger and more varied Android and IoT datasets. Besides, they insist on the necessity of conducting the model testing in actual resource-limited environments to check its scalability and durability.
^44^	Permissions, Intents, API-calls	98–99%	Random Forest, SVM, feature selection	This paper proposes an advanced Android malware detection framework that couples state-of-the-art feature engineering with machine learning classifiers for better accuracy and robustness against obfuscation. It enhances feature selection and dimensionality reduction to efficiently capture complex malware behavior.	The authors propose testing the model with larger and more diverse Android malware datasets and employing deep learning or hybrid analysis techniques in order to achieve greater adaptability and real-time detection in constantly changing threat landscapes.
^45^	permission sets, intent filters, API-calls	99%	Random Forest	The paper proposes a lightweight malware-detection framework, GuardDroid, optimized for Android-based IoT devices, which balances high detection accuracy with low resource usage. It also integrates explainable ML using SHAP values to enhance transparency and foster trust in IoT deployment contexts.	They suggest the deployment of GuardDroid in real-world heterogeneous settings of IoT devices to ensure scalability and robustness across different hardware. They also encourage further research on runtime dynamic behavior features and on-device implementation optimizations, such as model compression and edge inference.
^46^	Static Java code features (KeyCount dataset)	99.95%	Ensemble of RF, Bagging, Decision Tree + AOFS feature selection	AndroMD creates a large scale dataset, 3 static complementary datasets, optimal feature selection AOFS, ensemble detection of high accuracy, detects real malware and zero-day samples.	missing deep-learning FS comparison, no system-level/cloud performance, static-only design, future benchmarking needed
^47^	Static Android permissions	99.33% (real data), 98.28% (synthetic adversarial)	MLP, BernoulliNB, Passive-Aggressive	New permission-to-exploitation mapping; balanced 111,822-app dataset; selective incremental training; adversarial-robust evaluation; scalable detection	The authors should increase more datasets, add more malware families and permission changes, improve adversarial robustness, and integrate deeper hybrid analysis.

### Dynamic analysis techniques

Dynamic analyzes enable the detection of malware by executing applications in controlled environments such as emulators or sandboxes, from which their real-time behaviors can be observed. These behaviors capture system calls, memory usage, and network activity, in addition to process creation, and do so in a way that is much more difficult to obfuscate than static code. Thus it can effectively identify sophisticated threats evading static detection such as polymorphic malware or runtime repackaged malware.One of the most beneficial aspects of dynamic analysis is its resistance to code obfuscation and encryption, since it generates a log of the actions of an application during its execution rather than relying on its static structure. This applies more specifically to zero-day threats and malware utilizing runtime payloads or checks for execution conditions. Dynamic analysis, however, imposes a greater demand on resources as it requires an execution environment that can replicate or emulate the behavior of actual devices. This is particularly problematic in large-scale or resource-poor IoT deployments.

New models have used dynamic analysis to increase the classification accuracy of the analyzed malware and the resilience of the model against malware evasion. For example, EntropLyzer^48^ collected 141 dynamic features - API calls, memory usage, logcat entries, and process-level information-everbefore and after emulator reboots. This entropy-based behavioral profiling yielded 98.4% accuracy across 12 malware categories and 147 families using the CCCS-CIC-AndMal-2020 dataset. The article discussed the need to extend this test on actual devices in order to meet emulator-aware evasion techniques at par.BIR-CNN^32^, on the other hand, developed an Inception-Residual CNN architecture which was Batch-normalized and integrated static features, as well as dynamic features, for Android malware detection. When network traffic features were extracted, a dimensionality of 347 was achieved, whereas this approach’s classification accuracy reached 99.73%. This model succeeds because it gains access to deep representation-learning capabilities and also because it avoids overfitting with the regularization techniques within the model, such as batch normalization.

Nonetheless, despite the improvements these methods witnessed, some dynamic analysis models’ resource requirements and feature extraction times-are simply too high to apply in real-time IoT environments, where lightweight interpretable models are preferred^60^. Moreover, many solutions are not able to consider their functionality regarding malware detection in emulated or sandboxed environments: therefore, they tend to fail in the live environment. Table 2 summarizes the authors, contributions, methods, and performances of the major dynamic analysis methods for Android malware detection.

**Table 2:** A comparison of related work based on dynamic analysis techniques.

Ref.	Feature	Accuracy	ML techniques	Contribution	Recommendations
^48^	Memory, API calls, network, battery, logcat, and process features totaling 141 dynamic characteristics	98.4%	Decision Tree, Random Forest	Dynamic features are analyzed before and after rebooting the emulator, with entropy values calculated to track behavioral changes across 12 malware categories and 147 families, Using the CCCS-CIC-AndMal2020 dataset, the authors dynamically analyzed a large set of malware and benign samples to ensure robust classification and characterization.	The paper suggests extending the dynamic analysis to real devices, as some malware samples detect and avoid emulation environments, reducing the effective sample size, incorporating more feature types, particularly those that may bypass or obscure emulation detection, could enhance the detection model’s accuracy and reliability​
^32^	Network traffic data	99.73%	Random Forest (RF), Support Vector Machine (SVM), Decision Tree (DT), and Convolutional Neural Network (CNN)	Development of a BIR-CNN (Batch-normalized Inception-Residual CNN) model to classify Android malware, integrating inception-residual network modules with batch normalization to enhance learning and avoid overfitting, proposal of a 347-dimensional network traffic feature extraction method, improving feature comprehensiveness and model accuracy	Extend the BIR-CNN model for emerging Android software classification, identifying both benign and malicious applications, as well as categorizing new malware families, explore new datasets with diverse static and dynamic features for broader validation, enhancing the model’s generalizability across various network traffic profiles​
^33^	Permissions, API calls, intents	93.7%	SVM	the development of Sec-SVM, a secure-learning algorithm that enhances resilience against evasion attacks.	Applying secure-learning paradigms to other security tasks and continuing research on adversary-aware machine learning techniques, extending the secure-learning approach to other malware detection tasks and integrating dynamic analysis
^34^	Permissions and API calls	98.8%	SVM, MLP	The paper presents MLDroid, a web-based Android malware detection framework that effectively uses permissions and API calls to distinguish between benign and malicious apps.	exploring additional machine learning models and feature selection methods to further enhance malware detection capabilities, the application of MLDroid in real-world scenarios for detecting both known and unknown malware families efficiently.
^35^	Opcode, API features, and permission	98%	DNN	Android malware detection framework that utilizes multiple static features and a multimodal deep learning approach to improve detection accuracy	Exploring integrating dynamic features with the existing static feature-based framework to enhance detection capabilities, using the proposed multimodal deep learning appro3ach as it effectively improves the accuracy of Android malware detection​
^26^	API calls, permissions, intents	93.4%	Deep ANN	The paper introduces DeepAMD, a novel approach for detecting and identifying Android malware, which outperforms existing methods by enhancing detection accuracy on both static and dynamic layers​	Developing an online service to allow users to check whether an application is benign or malicious before downloading it​, using DeepAMP for effective and accurate detection and identification of Android malware, as it shows superior performance compared to other existing techniques
^19^	Permissions	99.97%	MLP, KNN and Random forest	The research evaluates 49 malware families using extensive datasets and demonstrates the effectiveness of machine learning classifiers in mobile malware detection.	The authors suggest further exploration of dynamic analysis techniques and broader datasets to improve detection accuracy, integrating anomaly-based intrusion detection systems with machine learning classifiers for enhanced mobile malware detection capabilities.
^45^	Runtime network-behavioral	98.6–99.1%	CNN, LSTM	In this paper, a collaborative threat intelligence framework for IoT is suggested that integrates blockchain for the secure sharing of threat data among the devices and applies machine learning models (CNN/LSTM among others) to the shared behavioral data for detection improvement across many devices. It showcases the capability of the decentralized ledger technology along with the collective intelligence to increase the detection of malware/attacks in the IoT significantly over the single-device solutions.	Implementing the framework in actual heterogeneous IoT networks (not just the dataset) so that its scalability and robustness can be validated. They also suggest investigating privacy-preserving sharing mechanisms, advanced ML models for evolving threats, and lightweight deployment appropriate for IoT devices with limited power further.

### Hybrid analysis techniques

Hybrid analysis involves combining static and dynamic analysis techniques to detect and classify IoT malware more effectively. Static analysis extracts features like permissions, API calls, and code structures, while dynamic analysis observes runtime behaviors, such as network activity and system interactions. By integrating both, hybrid analysis provides a comprehensive view, enhancing the detection accuracy and robustness^22^. Hybrid models are effective at solving some of the major flaws in each approach: analytic methods based solely on static analysis have trouble with obfuscated or encrypted code, fail to detect zero-day or polymorphic malware, and perform poorly at dynamic analysis, which is better at discovering behaviors at runtime. A hybrid model explicitly addresses these issues by combining the knowledge acquired from pre-execution analysis with the observed behavior at runtime, which is both more depth and breadth in characterization of malware. Table 3 provides a comparison of several hybrid detection frameworks. It shows the sets of features, classification algorithms, and their performance with benchmark datasets.

**Table 3:** A comparison of related work based on hybrid analysis techniques.

Ref.	Feature	Accuracy	ML techniques	Contribution	Recommendations
^49^	**Static Features**: Application permissions, code structures, API calls, and network addresses. **Dynamic Features**: Runtime behaviors, system calls, network traffic data, and memory utilization.	99.73%	Random Forest (RF), Support Vector Machine (SVM), Decision Tree (DT), and Convolutional Neural Network (CNN)	Development of a BIR-CNN (Batch-normalized Inception-Residual CNN) model to classify Android malware, integrating inception-residual network modules with batch normalization to enhance learning and avoid overfitting, proposal of a 347-dimensional network traffic feature extraction method, improving feature comprehensiveness and model accuracy	Extend the BIR-CNN model for emerging Android software classification, identifying both benign and malicious applications, as well as categorizing new malware families, explore new datasets with diverse static and dynamic features for broader validation, enhancing the model’s generalizability across various network traffic profiles​
^50^	**Static Features**: MAC addresses for device authentication and encryption keys. **Dynamic Features**: Real-time data from wearable devices for predictive modeling and anomaly detection.	99.99%	neural networks	Proposes a lightweight, hybrid mutual authentication framework using AES-128 encryption and MAC for secure communication in IoMT. Enables Big Data analytics and predictive modeling for early detection of health anomalies and improving patient outcomes.	Enhance the authentication process with **multi-factor security techniques**. Integrate advanced AI methods, such as **deep learning and large language models**, to improve predictive accuracy and adaptability.
^22^	**Static Features**: Permissions, code structures, API calls, and byte sequences from executable files. **Dynamic Features**: Behavioral patterns such as network operations, calls, and encryption activities	98.50%	deep CNN (SB-BR-STM) with ensemble classifiers like SVM, MLP, and AdaBoostM1.	Proposed a novel Deep Squeezed-Boosted and Ensemble Learning (DSBEL) framework for IoT malware detection. Developed an innovative SB-BR-STM CNN to capture diverse and minute malicious patterns. Enhanced classification performance using boosted features and ensemble ML classifiers for improved generalization.	Extend the framework for real-time IoT malware detection across online and Android platforms. Explore its robustness against zero-day attacks and its applicability in smart homes, healthcare systems, and industrial control systems.
^51^	**Static Features**: Pre-defined attack signatures such as specific patterns of data packets and thresholds for behaviors (e.g., packet drops). **Dynamic Features**: Behavioral patterns of zero-day attacks identified through unsupervised machine learning using Generative Adversarial Networks (GANs)	96%	GAN-based models	Proposed a trusted hybrid learning framework combining rule-based detection with GANs for robust detection of zero-day attacks. Introduced a Stackelberg trust game to improve collaboration and trust between distributed security engines.	Expand the framework to address more complex zero-day attacks by incorporating advanced machine learning models. Test and adapt the framework for large-scale real-world edge computing environments to ensure scalability and reliability.
^23^	**Static Features**: Permissions, API calls, application metadata, and network configuration. **Dynamic Features**: Runtime behaviors, system calls, memory usage, and network traffic patterns.	99%	Random Forest, SVM	Proposed a hybrid malware detection framework integrating static and dynamic analyses for IoT and Android systems. Highlighted vulnerabilities in IoT and Android architectures and recommended robust countermeasures for securing devices.	Extend the hybrid approach to cover emerging threats and zero-day vulnerabilities. Adapt the framework to evolving IoT ecosystems, enhancing scalability and robustness.
^52^	**Static Features**: Metrics derived from software properties, optimized using **Particle Swarm Optimization (PSO)** for dimension reduction. **Dynamic Features**: Behavioral data processed through **Multi-Layer Perceptron (MLP)** for fault prediction.	High accuracy	PSO, MLP	Proposes a hybrid fault prediction model combining PSO and MLP for IoT applications. Introduces a formal verification framework using Labeled Transition Systems (LTS) and the Process Analysis Toolkit (PAT) to validate the model.	Explore meta-heuristic algorithms for hybrid fault prediction to improve performance. Analyze additional functional properties, such as completeness, soundness, and fairness, for broader applicability.
^53^	network traffic data such as packet headers, protocol information, source and destination IP addresses, port details, and behaviors associated with attack types like DoS, worms, and reconnaissance.	96.98%	ANN, CNN, LSTM, and RNN	Developed the hybrid ACLR model for efficient botnet detection in IoT environments. Demonstrated superior performance over state-of-the-art models using comprehensive evaluation metrics (accuracy, precision, recall, F1-score, ROC-AUC, and PR-AUC).	Investigate reinforcement learning to automate training processes for improved adaptability. Enhance scalability and robustness for real-world applications in diverse IoT ecosystems.
^54^	**Static Features**: Electronic health records (EHR) such as blood pressure, sugar levels, cholesterol levels. **Dynamic Features**: Real-time sensor data including heart rate, body temperature, oxygen saturation, and respiratory rate.	99.45%	autoencoder neural network, ALO	Proposed a hybrid cryptographic framework (ALO-DHT) combining Ant Lion Optimization, Diffie–Hellman, and Twofish cryptographic techniques for enhanced data security and privacy. Validated the framework with performance metrics like accuracy, precision, recall, F1 score, and energy consumption, outperforming existing techniques.	Address computational overhead and improve scalability for resource-constrained edge AI networks. Explore compression mechanisms and lightweight cryptographic algorithms for real-time applications.
^29^	**Static Features**: Permissions and intents extracted from APK files, including SEND_SMS, RECEIVE_SMS, and ACCESS_NETWORK_STATE. **Dynamic Features**: Runtime behaviors such as system calls, data leaks, cryptographic usage, and network activity logs.	97%	Random Forest (RF), Support Vector Machines (SVM), Naive Bayes (NB), and TPOT	Proposed two hybrid frameworks, HybriDroid (hierarchical static and dynamic analysis) and cHybriDroid (simultaneous static and dynamic analysis), for robust Android malware detection. Demonstrated superior malware detection capabilities, especially against zero-day threats, by combining static and dynamic features.	Incorporate additional dynamic features like memory utilization and network statistics for broader coverage. Extend the framework to classify malware into specific families and enhance scalability for real-world applications.
^55^	**Static Features**: Predefined network traffic signatures and metadata from IoT devices. **Dynamic Features**: Real-time network traffic data, including packet behaviors during Mirai and BASHLITE botnet attacks.	100%	CNN, LSTM	Developed a hybrid CNN-LSTM model for precise detection and classification of IoT botnet attacks. Validated the system with real-world datasets from four IoT-connected security cameras, achieving state-of-the-art performance.	Optimize the system for scalability and real-time applications in large-scale IoT networks.
^56^	**static Features**: permissions, intents, opcodes, API calls **Behavioral Features**: extracted from application execution logs	99%	CNN + LSTM + pseudo-labeling	This paper introduces a semi-supervised hybrid deep learning framework (CNN + LSTM) for detecting Android malware with both labeled and unlabeled data. It effectively combines static and limited dynamic features to improve detection accuracy and adaptability against unknown malware variants.	the framework need to be broadened with instantaneous dynamic behavior observation and threat data specific to IoT in order to increase the generalization, the application of explainable AI techniques will likely result in better model transparency and confidence in the malware detection verdicts.

The methods utilized by these models for combining features of both static and dynamic sources are diverse. For instance, the BIR-CNN model^49^ utilizes inception-residual convolutional neural network units with high-dimensional traffic data to attain an accuracy of 99.73% in classifying malware. Analogously, DS-BEL^22^ utilizes a squeezed-boosted ensemble convolutional neural network for detecting low-level and contextual malicious activity, while HybriDroid^29^ utilizes concurrent static-dynamic analysis to strengthen the detection of zero-day vulnerabilities. Despite their high detection performance, many of these hybrids have certain weaknesses. Deep learning-based hybrids, like DS-BEL or BIR-CNN, often involve huge computations and require GPU-compliant environments, making them not suitable for power-constrained IoT devices this is proved in^56^ While the framework achieved high accuracy and increased adaptability compared to the traditional supervised methods, certain limitations still exist. The approach focuses mainly on Android application-level analysis, missing out on the integration of IoT-specific network traffic or device interaction patterns, which seriously constrains its applicability to broader IoT ecosystems. Additionally, this model represents a deep learning architecture, which introduces high computational complexity, making it hard to deploy in real time on lightweight IoT devices. In addition, many of them are lacking when it comes to explainability or interpretability, hence compromising trust in critical application fields such as healthcare or intelligent infrastructure^50,51,61^.Further, deplorability is mostly neglected. Few models have been tested on real IoT devices or publicly available datasets like Drebin or TUANDROMD or CCCS-CIC-AndMal-2020 or CIMD-2024.

This challenge is specifically addressed in our proposed framework, which uses an interpretable and lightweight Random Forest classifier with dual-ranked features that are trained using Information Gain and Gini Index, thus allowing real-time operability without compromising on accuracy and transparency. In summary, although hybrid approaches represent considerable progress in Android malware detection, it is still a difficult task to build systems that are effective, interpretable, and scalable. This work proposes an effective hybrid detection framework that is tailored to real-world IoT settings, with improved accuracy while maintaining acceptable explainability and low resource usage.

## Proposed AndroidIoT security systems framework

This section introduces a novel hybrid malware detection framework for Internet of Things (IoT) environments based on the Android operating system. The system integrates aspects of both static and dynamic analysis using an optimized Random Forest (RF) classifier based on a dual feature ranking strategy using Information Gain and Gini Index to attain high accuracy, interpretability, and low-power edge deployment compatibility.

**Fig. 1:**
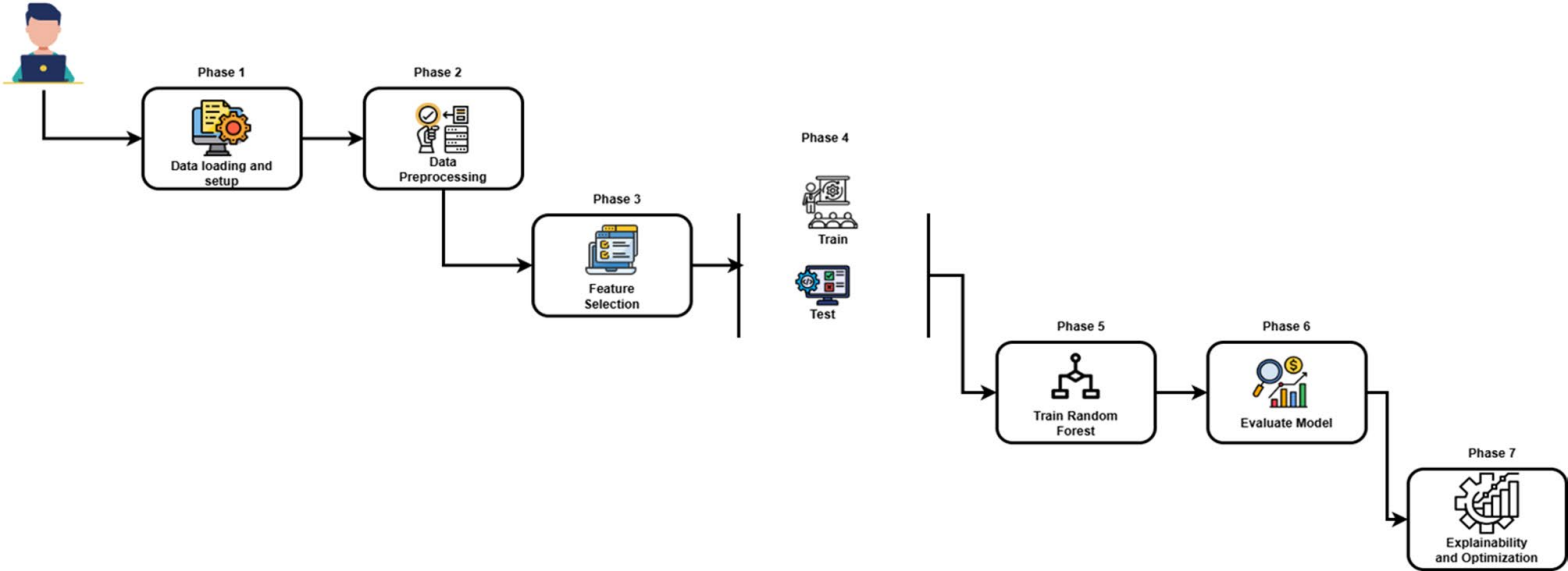
Framework of random forest classifier for AndroidIoT security systems.

The framework proposed in this paper seeks to address three basic challenges with Android malware detection: dealing with high-dimensional and noisy feature spaces, enabling efficient computation processes suitable for resource-constrained IoT settings, and maintaining interpretability to ensure security-critical decision-making in real-world applications. In an effort to address these goals, the architecture follows a structured seven-step framework that systematically guides data through processes of collection, preprocessing, feature selection, model training, and evaluation. The process illustrated in Fig. 1 ensures that the detection approach is efficient and transparent while being highly accurate in its classification process.

### Workflow phases of proposed AndroidIoT

There are seven different steps in the proposed system for detection of malware that collectively ensure good, easy, and efficient classification for Android-based Internet of Things scenarios.

#### *Phase 1*: data loading and setup

First of all, four common Android malware datasets, Drebin, TUANDROMD, CCCS-CIC-AndMal-2020 and CIMD-2024, are downloaded and prepared. The first one provides static permission and APIs information, and the second one provides both static and dynamic behavior information, such as memory and system call information. The datasets are downloaded, cleaned, and organized into Pandas DataFrames. Then exploratory data analysis (EDA) is carried out in order to examine the size of the data, determine the data type, and examine features distribution in order to check if data is unbalanced. The first inspection ensures that data is of good quality and consistent in order to build a solid foundation for additional preprocessing and modeling.

#### *Phase 2*: data preprocessing

The objective in this phase is to prepare the dataset for model training. Missing values are either imputed with appropriate defaults or removed depending on context and importance of the feature. Class labels such as ‘benign’ and ‘malware’ are converted into numbers in order for ML models to easily use these. The numeric features such as CPU and memory usage (very crucial in CIC-AndMal-2020) are scaled so that all features retain values within a similar range. The training performance is enhanced and biases against large feature values are eliminated.

#### *Phase 3*: feature selection

There is a two-step procedure in selecting the most important features and eliminating unnecessary ones in making the input space as descriptive as can be. The first phase is referred to as Information Gain and in it every feature is tested for its ability to decrease uncertainty in class labeling, particularly those features that can easily distinguish between different classes. The second phase is the Gini Index in which features are tested based on their ability to decrease confusion in a decision tree. Selecting the top features in both phases ensures that only the most key variables are selected. This enhances performance in classifications and makes it faster and easier to comprehend. We chose Information Gain and Gini Index instead of other ranking techniques (e.g., Chi-Square and Mutual Information) for our Android malware datasets to analyze mixed categorical/binary feature spaces. They are both computationally efficient and resilient to class imbalance, as they both leverage general splits made by Random Forest which adds consistency between the ranking of features and the final classifier. Our initial testing of Chi-Square and Mutual Information had a higher computational overhead and less stable rankings, particularly with an imbalanced Internet of Things (IoT) dataset.

#### *Phase 4*: Train-Test split

After identifying features, the data is divided into sets for training and testing in order to verify if and how efficiently the model performs. The conventional approach is applying the 80/20 rule in which 80% of data is trained against by the model and 20% is reserved for testing its performance. Stratified sampling is applied in order to ensure even distribution of both harmless samples and malware samples within sets. The phase is crucial if imbalances are in the dataset in order to prevent larger sets from impacting the performance measurement.

#### *Phase 5*: train random forest model

In this work, we have investigated a unified Random Forest malware detection engine on four benchmark datasets: Drebin, TUANDROMD, CCCS-CIC-2020, and CIMD-2024. Using grid search with five-fold cross-validation on shared hyperparameters (n_estimators ∈ {50–200} and max_depth ∈ {10–None}), all datasets achieved their best performance with 100 trees and a maximum depth of 20. Despite the diversity of the datasets in terms of feature types, which include static features in Drebin, hybrid static-behavioral features in TUANDROMD, dynamic execution traces in CCCS-CIC-2020, and mixed static-dynamic-network features in CIMD-2024, the Random Forest model consistently delivered strong accuracy, robust generalization, and low inference latency suitable for IoT and edge environments. That the optimum configuration is the same across such diverse feature spaces highlights the adaptability, noise resilience, and effectiveness of Random Forest in modeling complex malware behaviors.

#### *Phase 6*: evaluate model

Evaluate how good the model performs on the test set through a full set of evaluation metrics. The metrics are Accuracy, Precision, Recall, and F1-Score and represent different aspects on how the model is performing in classification. We also build a Confusion Matrix that reveals true positives, true negatives, false positives, and false negatives and allows us to visualize errors in classification. These metrics collectively give a good indication of how the model would perform in actual malware detection scenarios.

#### *Phase 7*: explainability and optimization

The final phase simplifies and improves things. We have rankings showing how significant each feature is based on the Random Forest model. These rankings indicate what features contributed most to making predictions. Security analysts and those who work with IoT would greatly benefit from this information since they require accurate predictions accompanied by reliable explanations. Meanwhile, we tune optimal model settings, preferably through Grid Search, to enhance items such as tree depth, number of estimators, and splitting rules. These tweaks also enhance both accuracy and resource efficiency in real-time use.

### Algorithm of the proposed AndroidIoT security

#### Algorithm 1

describes an end-to-end workflow for the AndroidIoT Security Framework proposed here, expertly combining static and dynamic analysis for optimal detection of malware through a Random Forest classifier. The process begins with loading and traversal of labeled data collection involving features of Android apps. Next, several key preprocessing steps are undertaken in the process, including filling in missing values, categorical encoding of variables for labeling purposes, and normalization of numeric features for uniform representation. The process uses a dual-ranking feature reduction process through both Information Gain and Gini Index for identification of crucial features while simultaneously reducing dimensions. The processed data is then split into a training set and a testing set through a stratified 80:20 split while carefully preserving the distribution of classes. A Random Forest model is initialized through a predetermined number of trees and depth and trained on identified features. Trained model performance is tested on the test set using various performance metrics in terms of accuracy, precision, recall, and F1-score while a confusion matrix provides insightful information about effectiveness of the classification. Finally, feature importance analysis and model tuning are included through grid search for improving interpretability as well as predictive power. The algorithm embodies an efficient, explainable, and light design, best suitable for real-time identification of malicious apps in the Android-based Internet of Things environment. So In order to ensure reproducible feature selection as shown in Table 4, independent computation of IG and GI scores for all features was performed. Each feature had a normalized score in both ranking lists, and a unified hybrid ranking score was obtained by taking the average of the normalized values of IG and GI. The final selected feature set consisted of the top-k features based on this hybrid score, ensuring that features that were consistently important across both criteria were prioritized. This reduces bias towards one ranking method and enhances robustness in selecting discriminative features.

**Table 4:** Lists the final random forest hyperparameters selected.

Hyperparameter	Selected Value	Description
n_estimators	100	Selected for balance between stability and speed
max_depth	20	Prevents overfitting while keeping model expressive
min_samples_split	2	Best-performing value during tuning
min_samples_leaf	1	Ensures deeper tree splits when needed
max_features	sqrt	Standard RF practice; best CV performance
Bootstrap	gini	Outperformed entropy in cross-validation
Criterion	true	Ensures variance reduction
class_weight	balanced” (if used)	Improves performance on slightly imbalanced datasets

**Algorithm 1:**
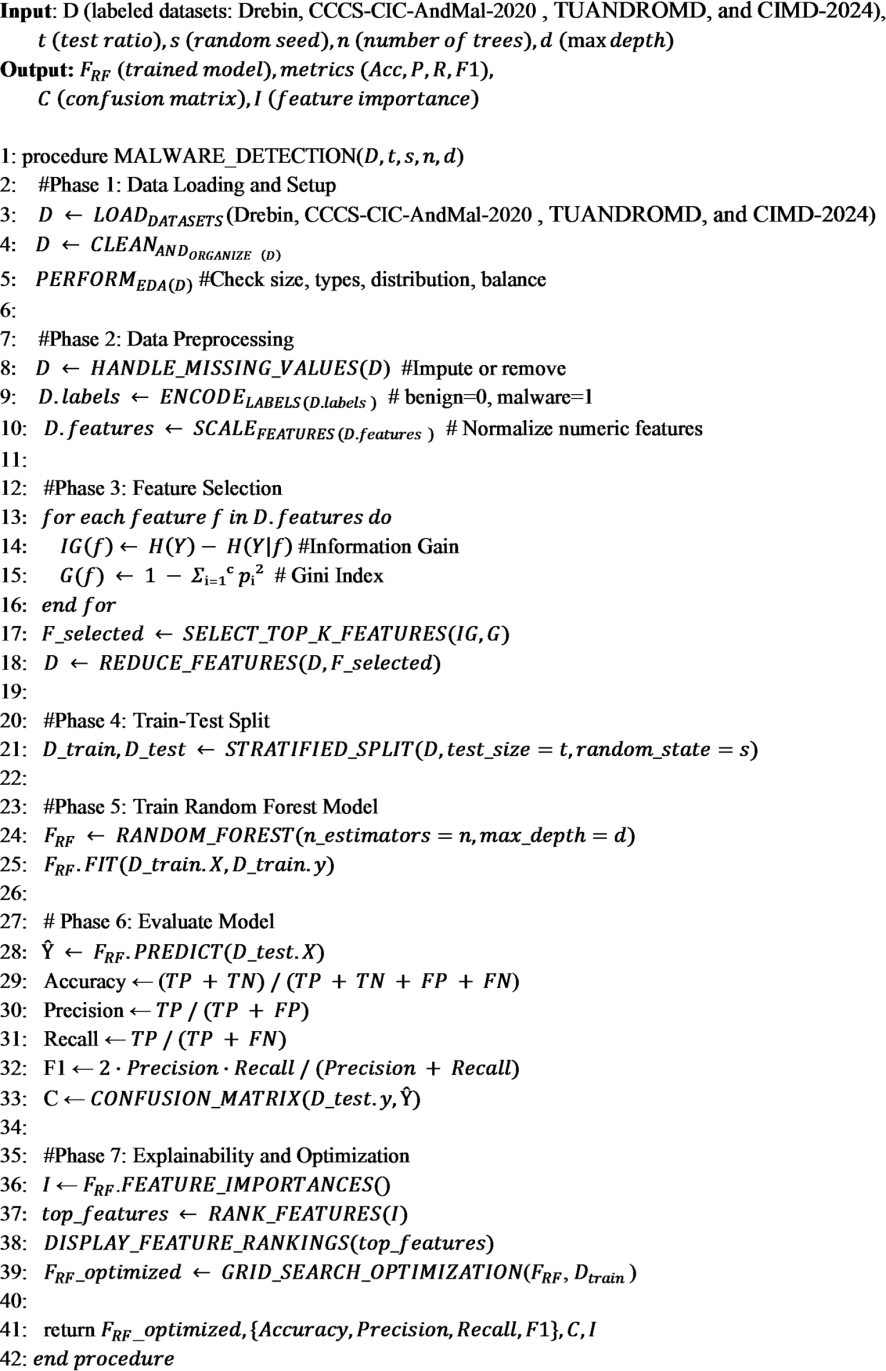
Hybrid android-IoT malware detection framework using random forest.

## Experimental evaluation

For the purpose of validating the hybrid detection framework, experiments were conducted rigorously with four well-known Android malware datasets: **Drebin** (static features), **TUANDROMD** (static features) and **CCCS-CIC-AndMal-2020** (hybrid static and dynamic features) and **CIMD-2024** (dynamic features). The four datasets had distinct merits in querying the system’s robustness across the two separate analysis paradigms. Static analysis offers a computationally efficient approach to Android malware detection by examining application code without execution. This contrasts with dynamic analysis, which observes runtime behavior.

### Dataset overview and feature characteristics

The **Drebin dataset** facilitates lightweight, explainable malware detection via static analysis. It consists of 15,036 Android applications, divided into malware (5,560 samples) and benign applications (9,476 samples). It is mainly for Android malware detection research, and the 216 extracted features represent different aspects of application behavior. It employs feature extraction, encoding characteristics such as requested permissions, API calls, intents, and network addresses into a vector space suitable for machine learning classification^11^.This static approach, exemplified by Drebin, avoids the overhead of application execution, making it particularly well-suited for resource-constrained Internet of Things (IoT) devices^9^. The combination of the Drebin dataset with a Random Forest classifier enables accurate identification of malware patterns in Android IoT environments, minimizing both false positives and computational demands as described in first dataset results. Hybrid analysis integrates static and dynamic analysis to improve malware detection by examining both code-level attributes and real-time execution behavior. Static analysis detects permissions, API calls, and intents, while dynamic analysis captures runtime activities such as network communication and system modifications. This approach overcomes limitations like obfuscation, making it more robust than using either method alone^49^. Also using the **TUANDROMD** dataset is a well-known benchmark for Android malware detection and consists of 4,465 applications, including 3,365 malicious and 1,000 benign samples. It includes 241 statically extracted features about different kinds of application characteristics. In this paper, TUANDROMD will be used to evaluate the generalization of models on unseen malware samples of Android IoT environments^57^. Using the **CCCS-CIC-AndMal-2020** dataset, our framework applies this hybrid technique to classify malware efficiently. It is a collection of 53,439 Android applications with 145 extracted features, which focus on detecting malware based on memory usage and system resource consumption as well as app interactions. It contains important attributes such as heap allocation, Inter Process Communication (IPC) and memory statistics, and is useful for dynamic behavior analysis. This dataset is frequently employed in machine learning-based malware detection to detect stealthier, more resource hungry malware. The dataset provides both static and dynamic features, allowing comprehensive malware detection. This enhances accuracy, improves detection resilience, and strengthens Android IoT security systems as described in second dataset results. Also the **CIMD-2024** dataset comprises a real-world, time-series dataset developed for multi-class malware detection and network anomaly analysis in Industrial Internet of Things environments. It ranges from network activity and system performance metrics over five years (November 2019 to December 2024) and captures detailed hourly logs from diverse IIoT devices such as sensors, actuators, cameras, and gateways. Each sample of the dataset contains a feature set of around 40 features, categorized into network traffic, statistical, payload, and system-level features, which are labeled for multi-class classification, including but not limited to Benign, Ransomware, Spyware, Botnet, Trojan, and Worm. It is structured to facilitate advanced cybersecurity research, including AI-based intrusion detection and federated learning applications, though it contains a significant class imbalance that may challenge traditional machine learning models^30^.

### The features importance analysis

#### Drebin dataset

**Fig. 2:**
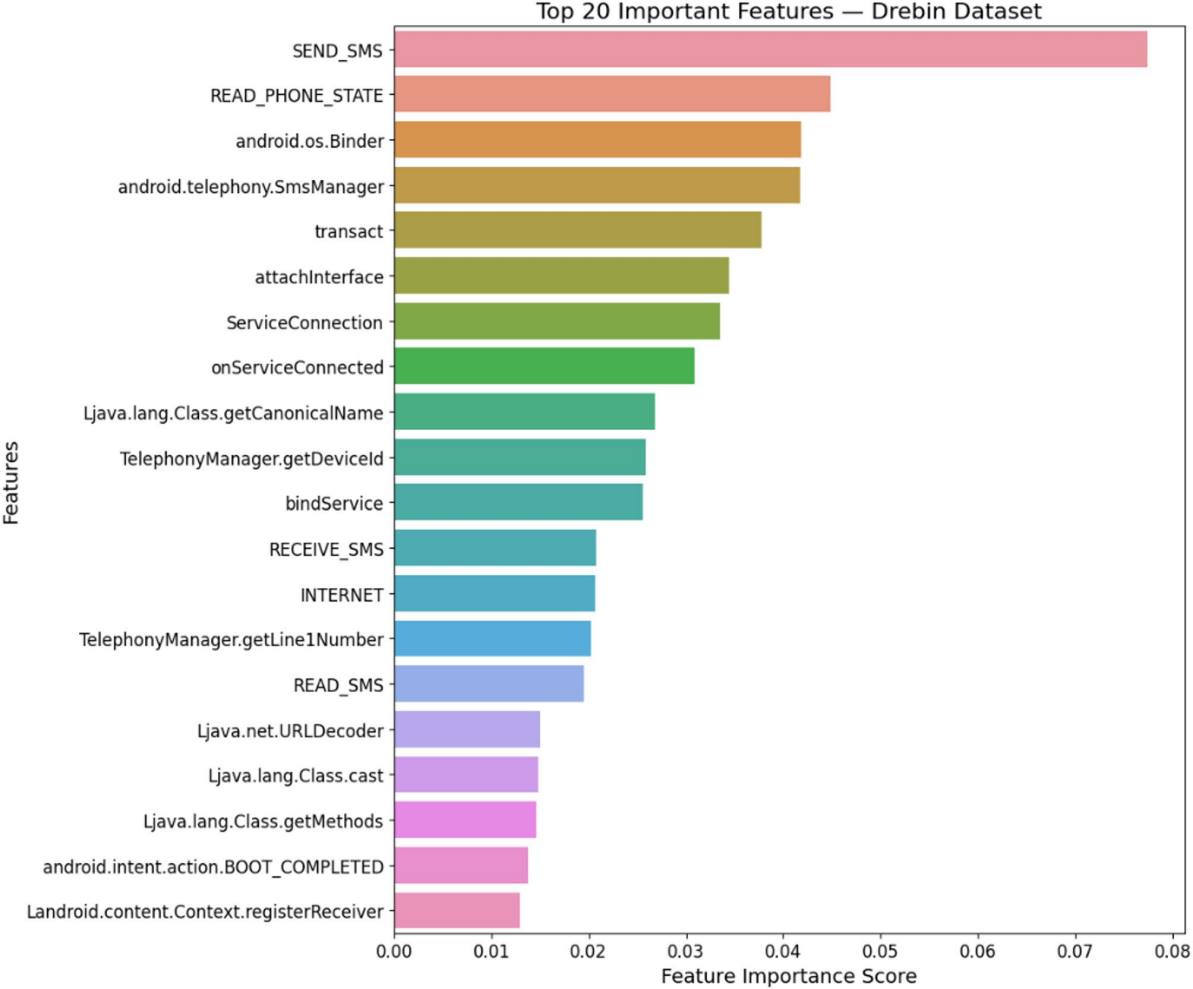
Feature importance of Drebin dataset.

Using the Random Forest classifier on the Drebin dataset reveals the varying contributions of static features in detecting Android malware. The visualization demonstrates that permissions, API calls, intents, and network addresses play a crucial role in distinguishing malicious applications from benign ones. Features with higher importance scores, such as restricted API calls and network connections, indicate strong correlations with malware behavior, while lower-scoring features contribute minimally to classification. The visualization demonstrates the varying contributions of static features, guiding optimization for improved classification performance as shown in Fig. 2.

#### TUANDROMD dataset

**Fig. 3:**
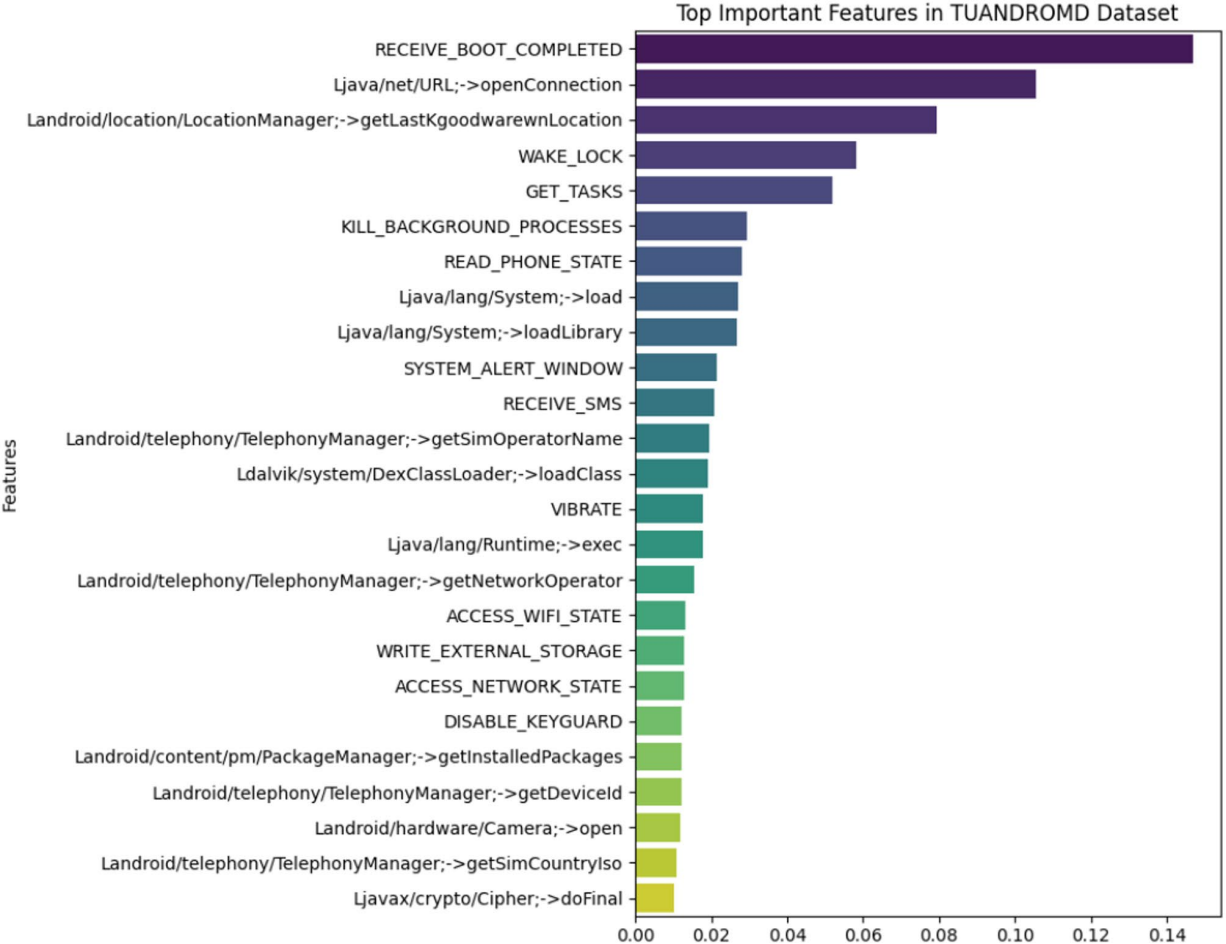
Feature importance of TUANDROMD dataset.

The feature importance analysis carried out using the Random Forest classifier on the TUANDROMD dataset points to the important roles of some Android permissions and API-related calls in distinguishing malware from benign applications. Permissions like RECEIVE_BOOT_COMPLETED and API-related features, such as Ljava/net/URL;-> openConnection, are the top static features, which insinuate that these capabilities are highly associated with malicious behavior.as shown in Fig. 3.

#### CCCS-CIC-AndMal-2020 dataset

**Fig. 4:**
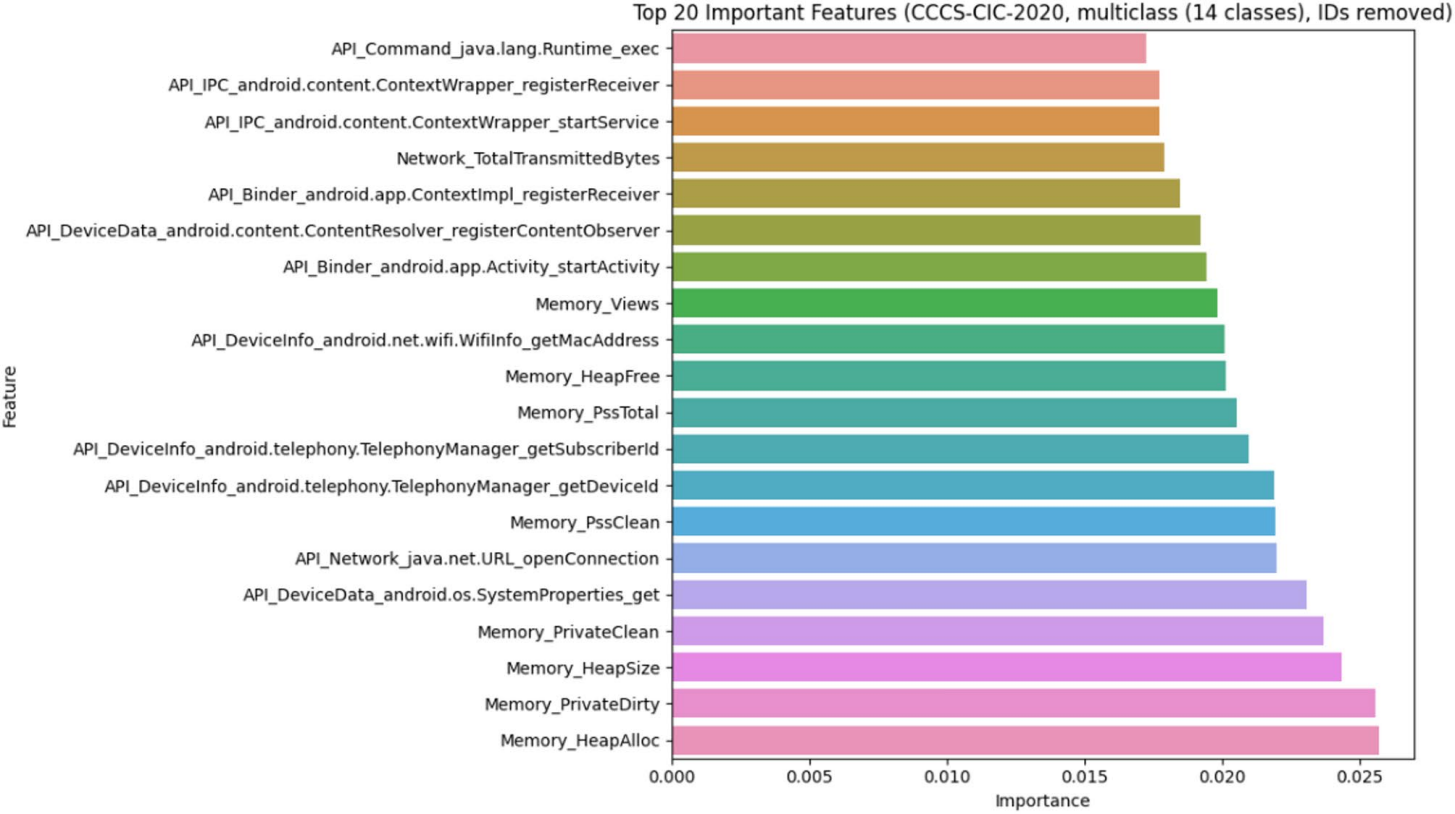
Feature importance of CCCS-CIC-AndMal-2020 dataset.

Using the Random Forest classifier on the dataset highlights the importance of both static and dynamic features in malware detection. Key attributes like API calls, network traffic, and behavioral patterns strongly correlate with malicious activity. The visualization demonstrates their varying contributions, optimizing hybrid analysis for improved classification performance, as shown in Fig. 4.

#### CIMD-2024 dataset

**Fig. 5:**
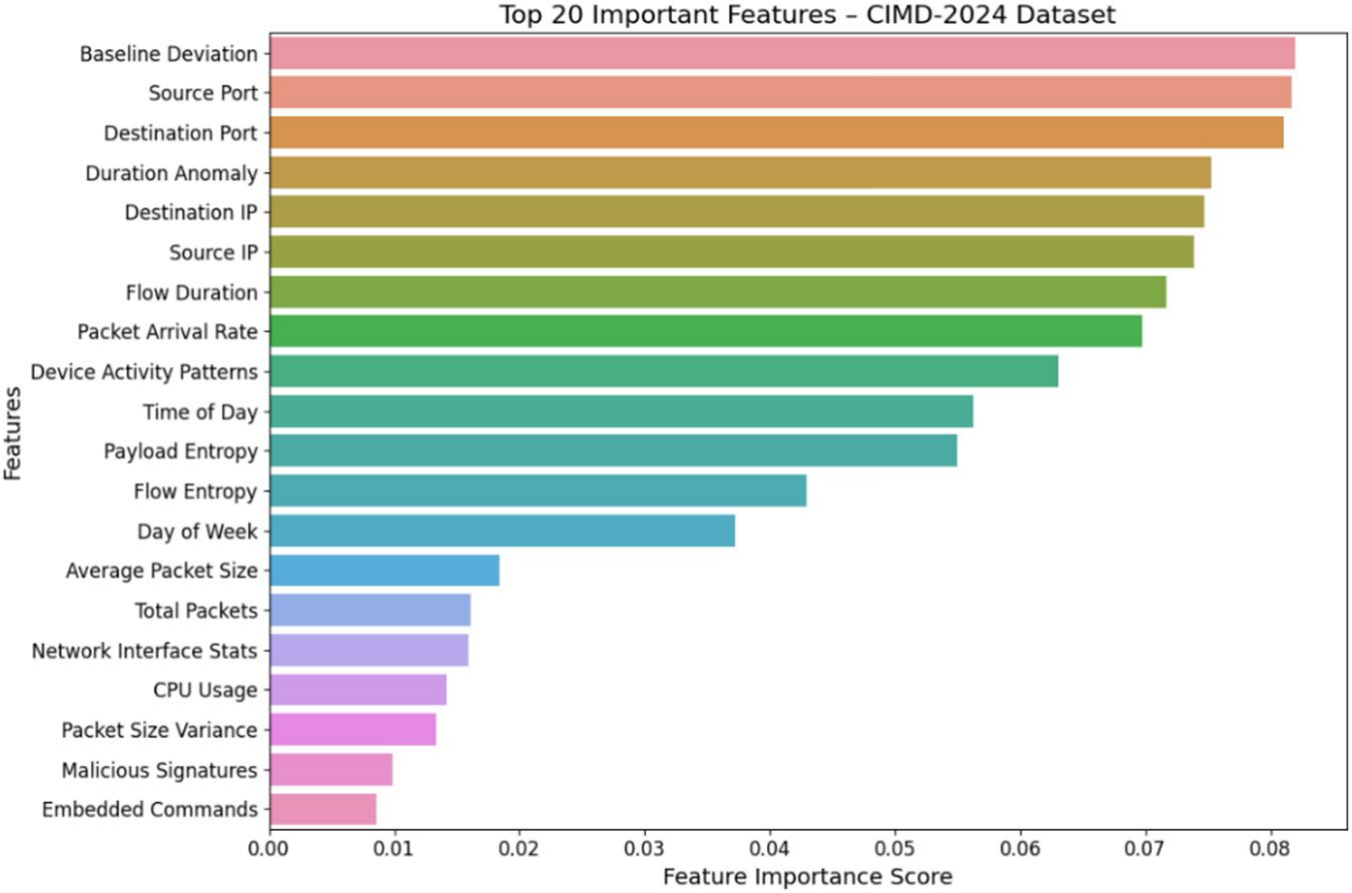
Feature importance of CIMD-2024 dataset.

The most informative features on the classification of IoT malware are mostly network-based and behavioral in nature according to the CIMD-2024 dataset. The most contributing features to the model’s decision are baseline deviation, source/destination port, and timestamp, which suggests that abnormal traffic patterns and variations in timing are strong indications of malicious activity. Low-ranking features such as Memory Usage or Total Bytes have little impact, and the network behavior dominates for malware detection in this dataset as shown in Fig. 5..

### Evaluation metrics and performance assessment

For a comprehensive evaluation of our framework, we utilized standard metrics such as accuracy, precision, recall, F1-score, and confusion matrix. Using Python’s robust data science libraries, we analyzed classification accuracy to measure the effectiveness of our Random Forest-based model. The detailed description of performance evaluation metrics can be shown in^58^, Standard metrics calculated as the following:

- **True Positive (TP)**: The number of **malicious** records correctly classified as **malicious**.
- **True Negative (TN)**: The number of **benign** samples correctly identified as **benign**.
- **False Positive (FP)**: The number of **benign** samples incorrectly classified as **malicious**.
- **False Negative (FN)**: The number of **malicious** records mistakenly classified as **benign**.
  1. **Accuracy**: is the ratio of correctly predicted samples to the total number of samples.

1 $$\:\mathbf{A}\mathbf{c}\mathbf{c}\mathbf{u}\mathbf{r}\mathbf{a}\mathbf{c}\mathbf{y}\:=\:\frac{\boldsymbol{T}\boldsymbol{P}+\boldsymbol{T}\boldsymbol{N}}{\mathbf{T}\mathbf{P}+\mathbf{T}\mathbf{N}+\mathbf{F}\mathbf{P}+\mathbf{F}\mathbf{N}}$$

2. **Precision**: is the proportion of correctly classified malicious applications to the total number of applications predicted as malicious.

2 $$\:\mathbf{P}\mathbf{r}\mathbf{e}\mathbf{c}\mathbf{i}\mathbf{s}\mathbf{i}\mathbf{o}\mathbf{n}\:=\:\frac{\boldsymbol{T}\boldsymbol{P}}{\mathbf{T}\mathbf{P}+\mathbf{F}\mathbf{P}}$$

3. **Recall**: is the proportion of correctly identified instances within the total records of each class. It is also referred to as the **True Positive Rate (TPR)**.

3 $$\:\mathbf{R}\mathbf{e}\mathbf{c}\mathbf{a}\mathbf{l}\mathbf{l}\:=\:\frac{\boldsymbol{T}\boldsymbol{P}}{\mathbf{T}\mathbf{P}+\mathbf{F}\mathbf{N}}$$

4. **F1-SCORE**: score shows the correlation between recall and precision.

4 $$\:\mathbf{F}1-\mathbf{S}\mathbf{C}\mathbf{O}\mathbf{R}\mathbf{E}\:=\:\frac{2\boldsymbol{*}\boldsymbol{T}\boldsymbol{P}}{2\mathbf{*}\mathbf{T}\mathbf{P}+\mathbf{F}\mathbf{P}+\mathbf{F}\mathbf{N}}$$

5. **Matthews Correlation Coefficient (MCC)** : measure of the quality of a classification model, It is one of the most reliable metrics in imbalanced datasets.

$$\:\mathbf{M}\mathbf{C}\mathbf{C}\:=\:\left(\:\right(\mathbf{T}\mathbf{P}.\:\boldsymbol{T}\boldsymbol{N})\:\--\:(\mathbf{F}\mathbf{P}.\:\mathbf{F}\mathbf{N}\left)\right)/\:(\sqrt{(\mathbf{T}\mathbf{P}+\mathbf{F}\mathbf{P})(\mathbf{T}\mathbf{P}+\mathbf{F}\mathbf{N})(\mathbf{T}\mathbf{N}+\mathbf{F}\mathbf{P}(\mathbf{T}\mathbf{N}+\mathbf{F}\mathbf{N})}$$

### Classification report

**Fig. 6:**
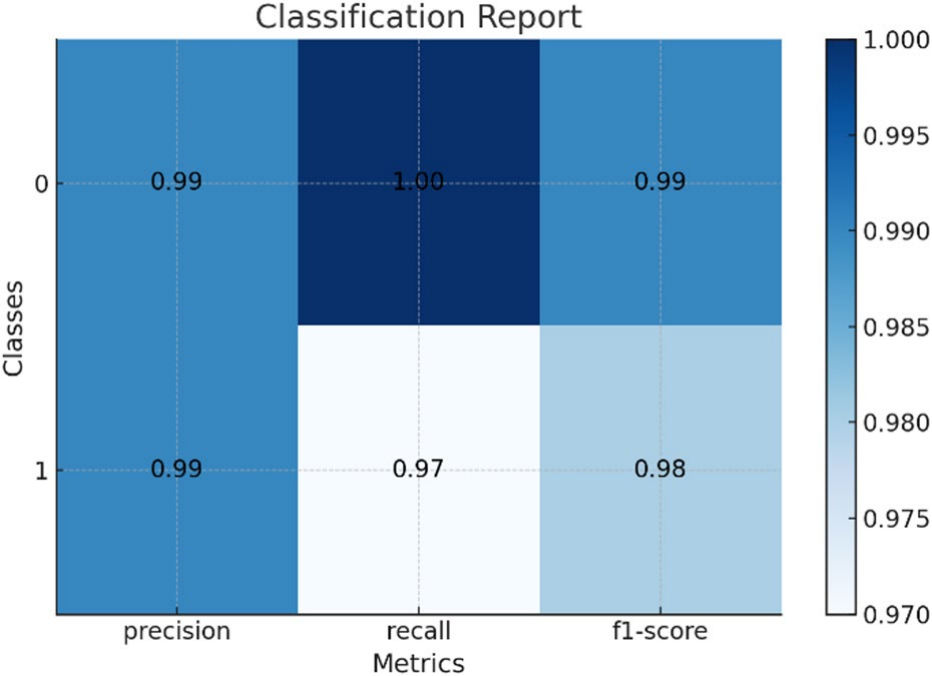
Classification report of Drebin dataset.

**Fig. 7:**
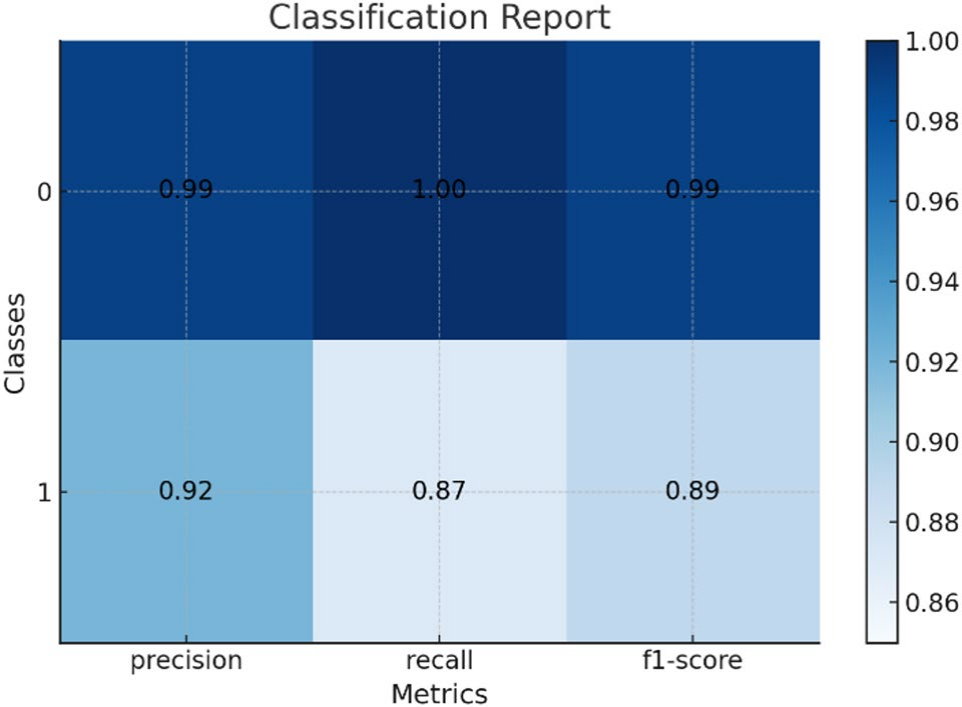
Classification report of TUANDROMD dataset.

**Fig. 8:**
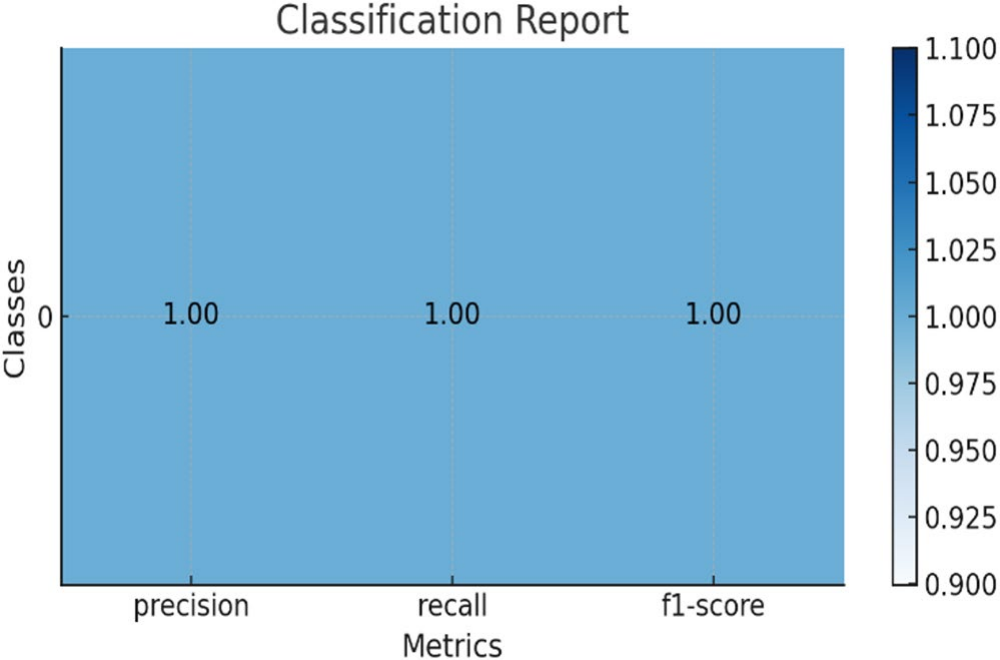
Classification report of CCCS-CIC-AndMal-2020 dataset.

**Fig. 9:**
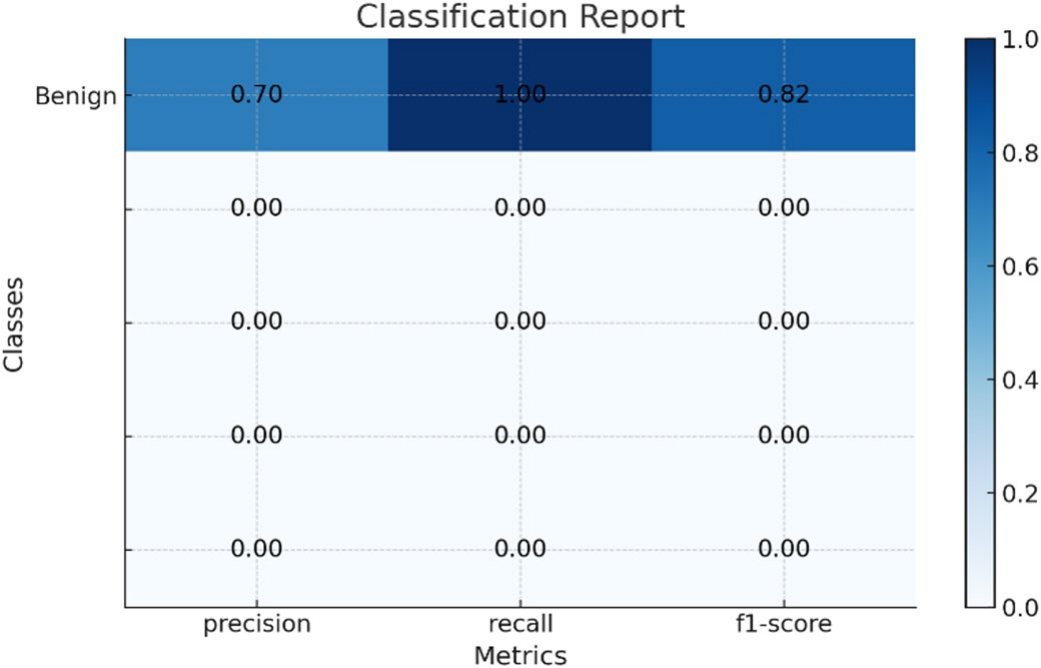
Classification report of CIMD-2024 dataset.

Each dataset as shown in Table 5 entry includes the following information, here classification report for the results of datasets shown in Fig. 6 for Drebin dataset and Fig. 7 for TUANDROMD and Fig. 8 for CCCS-CI C-AndMal-2020 and Fig. 9 for CIMD-2024 :

**Table 5:** Classification reports result on the datasets.

Dataset name	Features	Composition (malware/benign)	Accuracy (%)	Precision (%)	Recall (%)	F1 Score (%)	MCC (%)
Derbin	Static	5,560/9,476	99.03%	99%	98%	99%	97.43%
TUANDROMD	Static	3,365/1,000	98.66%	93.9%	93.9%	93.9%	86.61%
CCCS-CIC-AndMal-2020	Hybrid	28,380/25,059	100%	100%	100%	100%	49.69%
CIMD-2024	Dynamic	14,946/10,475	70.09%	12.0%	17.0%	14.0%	9.01%

### Confusion matrix

**Fig. 10:**
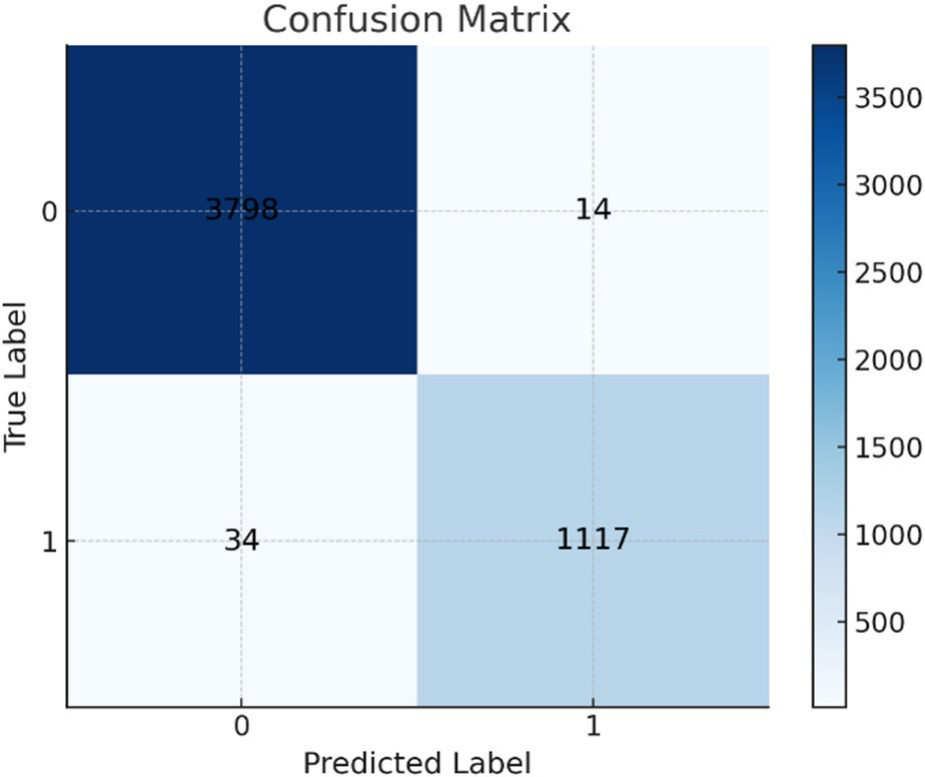
Confusion matrix of Drebin dataset.

**Fig. 11:**
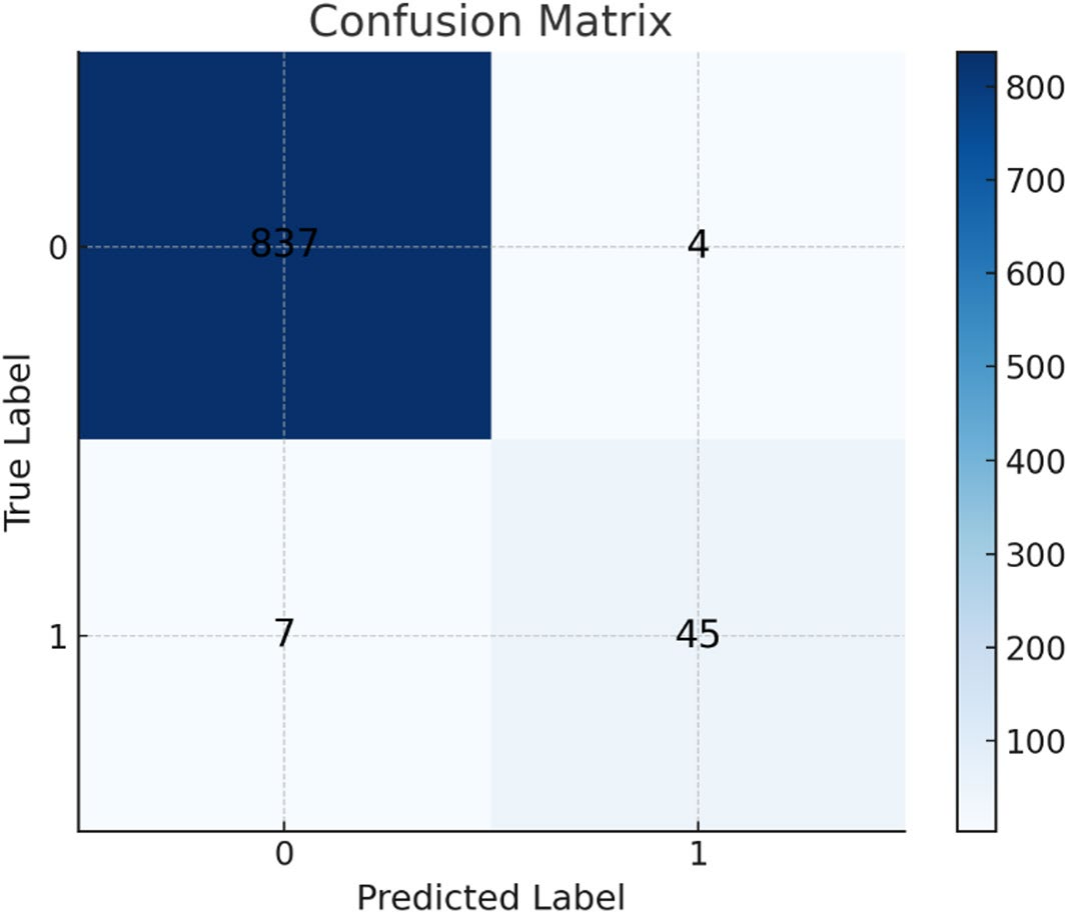
Confusion matrix of TUANDROMD dataset.

**Fig. 12:**
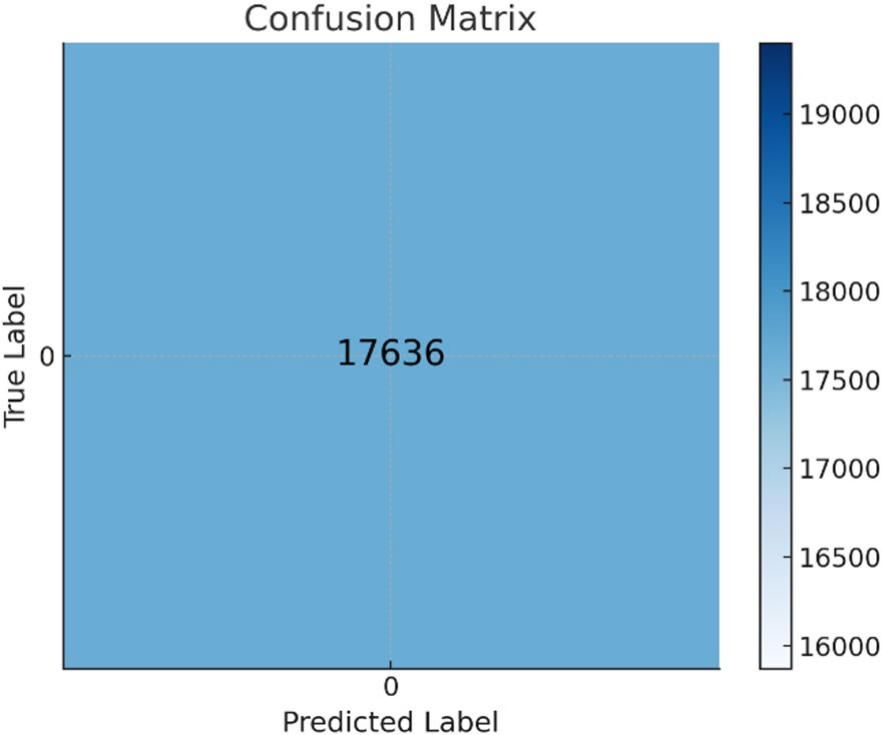
Confusion matrix of CCCS-CIC-AndMal-2020 dataset.

**Fig. 13:**
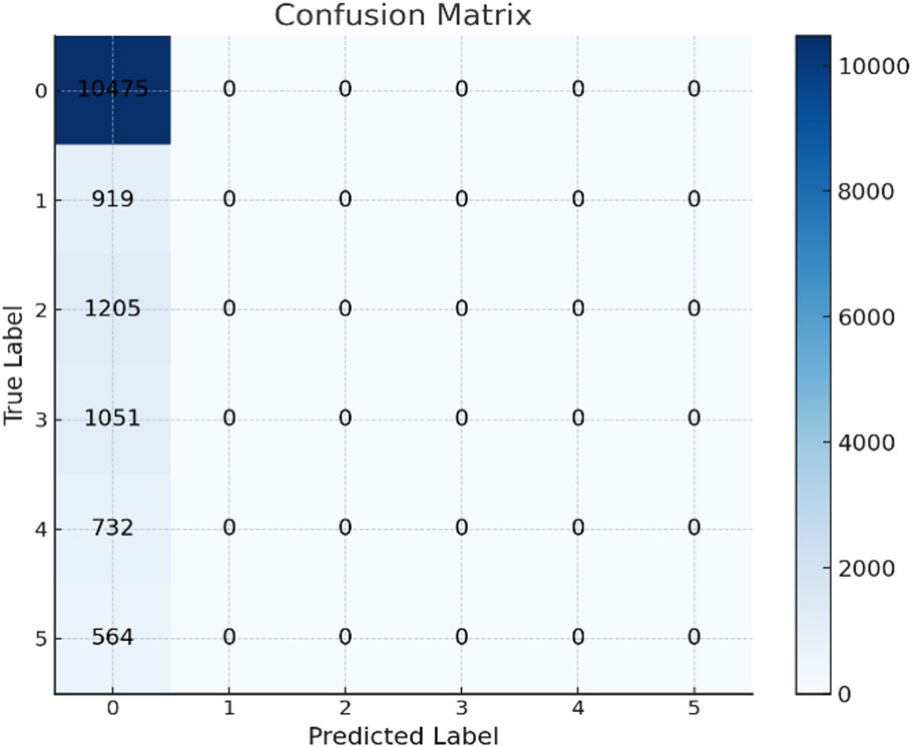
Confusion matrix of CIMD-2024 dataset.

It is a tool used to evaluate the overall performance of a classification model, applicable to both binary and multi-class classifications. It provides insights into the results, helping to assess model effectiveness. The confusion matrix of the datasets shown in Fig. 10 for Drebin dataset, the confusion matrix of TUANDROMD shown in Figs. 11 and 12 shown the confusion matrix CCCS-CIC-AndMal-2020 and Fig. 13 shown the confusion matrix CIMD-2024:

### Cross-Validation strategy for model evaluation

Cross-validation is a statistical evaluation technique that assesses how well a machine-learning model generalizes to completely unseen data. Thus, it does not only use one single train-test split but repeatedly partitions the entire data into several folds, securing that every instance is used for training and testing at a different iteration. For my framework, a k-fold stratified cross-validation procedure will be done where the data is divided into k equal-sized subsets with regard to keeping the original class distribution intact. Thus, during every run of the k-fold, the model will be trained on the (k-1) folds and evaluated on the remaining fold. The overall performance is returned as an average across all folds. This resampling technique gives a more robust, reliable estimation of accuracy, precision, recall, and F1 score with reduced risk of overfitting; hence, the evaluation does not depend on any random splitting^59^.In the framework, The mean score gives the model’s average performance across all folds, while the standard deviation represents the stability of that performance. With a low standard deviation, it means that the model behaves consistently across different splits; hence, the evaluation is more reliable, especially when dealing with imbalanced IoT malware datasets. So to ensure that the model performance was stable and that no overfitting or data leakage took place across all the folds. The very minimal variance in the accuracy and macro-F1 testifies to the robustness of this framework. All preprocessing steps (imputation, encoding, and feature scaling) were performed inside the cross-validation loop using scikit-learn pipelines. This guarantees that statistics (e.g., medians, encodings) are learned only from the training fold and then applied to the validation fold, thus avoiding any data leakage across folds.

#### Drebin dataset results

To obtain a robust estimate of the generalization ability of the proposed framework on the Drebin dataset as shown in Tables 5 and 6-fold stratified cross-validation was utilized. The model achieved an average accuracy of 98.78% with a very small standard deviation of ± 0.27%, which proves that its performance is very stable across folds. Besides, the macro-precision, macro-recall, and macro-F1 are 0.9888, 0.9849, and 0.9868, respectively, with a small variance for each. These results confirm that the framework not only achieves very good overall detection performance but also holds highly consistent detection capability for both benign and malicious classes, which is very important for reliable Android IoT malware detection.

**Table 6:** Cross validation results for Drebin.

Metrics	Mean Score	Standard Deviation
**Accuracy**	0.9878	± 0.0027
**Precision**	0.0988	± 0.0022
**Recall**	0.9849	± 0.0037
**F1-Score**	0.9868	± 0.0029

#### CCCS-CIC-AndMal-2020 dataset results

The experimental results show that the proposed framework is strong and stable in terms of performance on the CCCS-CIC-AndMal-2020 dataset as shown in Table 7 using 5-fold stratified cross-validation. The model achieves a high mean accuracy of 93.03%, reflecting good overall detection capability for the different malware categories. The macro-precision score is 93.82%, showing the framework’s ability to keep the number of false positives small for all classes, while the macro-recall is 85.72%, indicating that most malware families are correctly identified despite class imbalance. The macro-F1 score is 89.12%, thus balancing the precision and recall well. The remarkably low standard deviations for all metrics demonstrate that the model is quite consistent across different folds and is not biased toward the specific partitions of the data, showing good generalization to unseen samples.

**Table 7:** Cross validation results for CCCS-CIC-AndMal-2020.

Metrics	Mean Score	Standard Deviation
**Accuracy**	0.9866	± 0.0032
**Precision**	0.9849	± 0.0045
**Recall**	0.9832	± 0.0048
**F1-Score**	0.9840	± 0.0043

#### TUANDROMD dataset results

The performance of the proposed framework on TUANDROMD as shown in Table 8was very consistent and robust under 5-fold cross-validation, with an average accuracy of 0.9866 that had very small variance across folds ± 0.0032. In the same vein, the macro-precision was 0.9849 ± 0.0045, macro-recall was 0.9832 ± 0.0048, and macro-F1 score was 0.9840 ± 0.0043-all very stable and thus indicative of reliable performance on different data partitions. In fact, its narrow standard deviations for all metrics confirm that the framework is not sensitive to fold selection and maintains good generalization capability. These results indicate that the model can reliably learn from discriminative features in this dataset and will not suffer from any overfitting or random fluctuation in performance. Overall, the cross-validation results validate the robustness and stability of the proposed detection framework.

**Table 8:** Cross validation results for TUANDROMD.

Metrics	Mean Score	Standard Deviation
**Accuracy**	0.9866	± 0.0032
**Precision**	0.9849	± 0.0045
**Recall**	0.9832	± 0.0048
**F1-Score**	0.9840	± 0.0043

#### CyberTec IIoT malware dataset (CIMD-2024) dataset results

The proposed framework was tested on the highly imbalanced CIMD-2024 industrial IoT malware dataset as shown in Table 9 with a 5-fold cross-validation strategy in order to ensure reliable generalization. Through such strategy, an accuracy of 0.6982 has remained very stable on average, hence proving the model is systematically correctly classifying most of the samples across all folds. At the same time, macro-precision, macro-recall, and macro-F1 remain low, equal to 0.1450, 0.1370, and 0.1370 respectively, revealing the strong influence of class imbalance. Since the dataset is dominated by benign traffic, its classifier seems to easily obtain a high overall accuracy value but struggles to capture minority malware classes. Each fold has a very similar distribution and model behavior, as reflected by zero standard deviation across folds, confirming that poor performance on minority classes is systematic and not fold-specific. These findings clearly point to the use of imbalance-handling strategies-resampling, class-weighting, or feature-level balancing-as a clear next step of this work to improve malware class detection.

**Table 9:** Cross validation results for CIMD-2024.

Metrics	Mean Score	Standard Deviation
**Accuracy**	0.6982	0.0000
**Precision**	0.1450	0.0000
**Recall**	0.1370	0.0000
**F1-Score**	0.1370	0.0000

### Precision-Recall and ROC-AUC evaluation

Because many of the IoT malware datasets suffer from class imbalance, several more metrics have been computed to validate performance beyond accuracy. The Receiver Operating Characteristic (ROC) curve and its Area Under the Curve (AUC) were used to assess the classifier’s capability to distinguish between benign and malicious behavior across varying thresholds. Moreover, the Precision-Recall (PR) curve, which is more informative in the case of imbalanced datasets, has been computed to evaluate the quality of the model predictions of the positive class. These high values of AUC and PR-AUC reported across datasets confirm that the framework maintains strong discriminative power and robust generalization without relying only on accuracy.

#### Drebin dataset evaluation

**Fig. 14:**
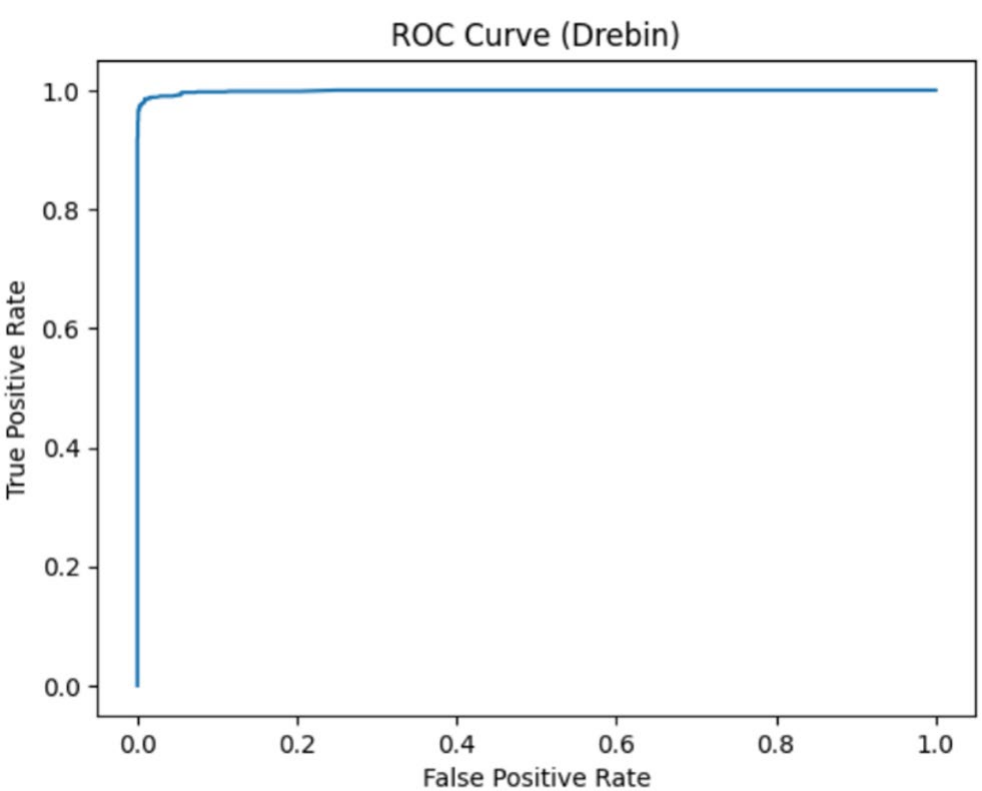
The ROC curve results on Drebin.

**Fig. 15:**
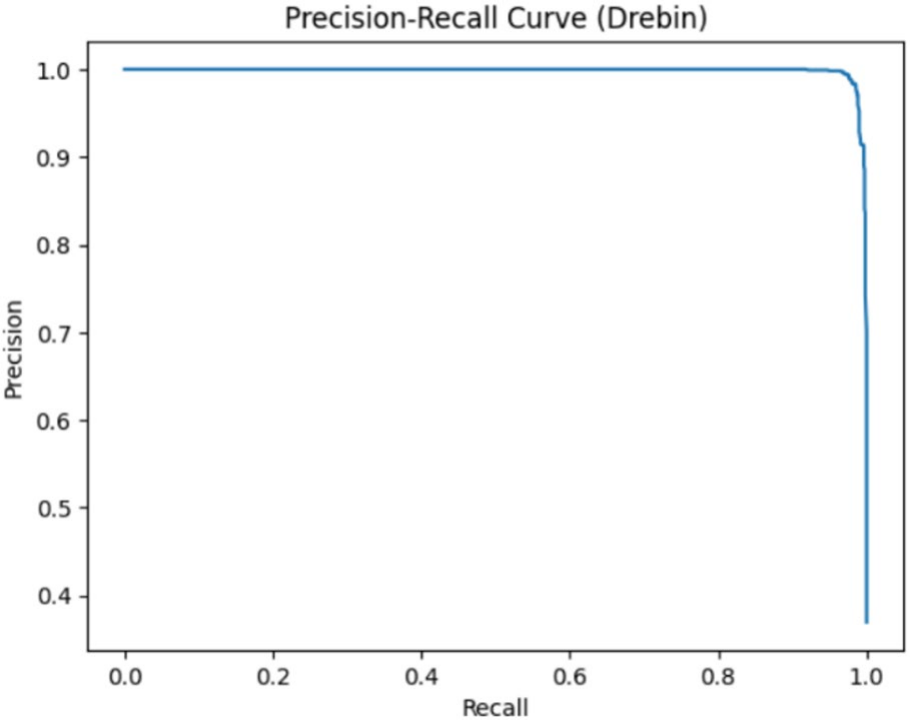
The PR curve results on Drebin.

The ROC and Precision-Recall curves for the Drebin dataset present AUC values above 0.998 as shown in Fig. 14, reflecting excellent class separability and indicative of consistent detection capability across various thresholds. The high PR-AUC as shown in Fig. 15 indicates that the model keeps extremely low false-positive and false-negative rates, confirming its reliability even under class imbalance. These curves confirm that there is no overfitting or data leakage in the model’s performance, together with the results of k-fold cross-validation.

#### TUANDROMD dataset evaluation

**Fig. 16:**
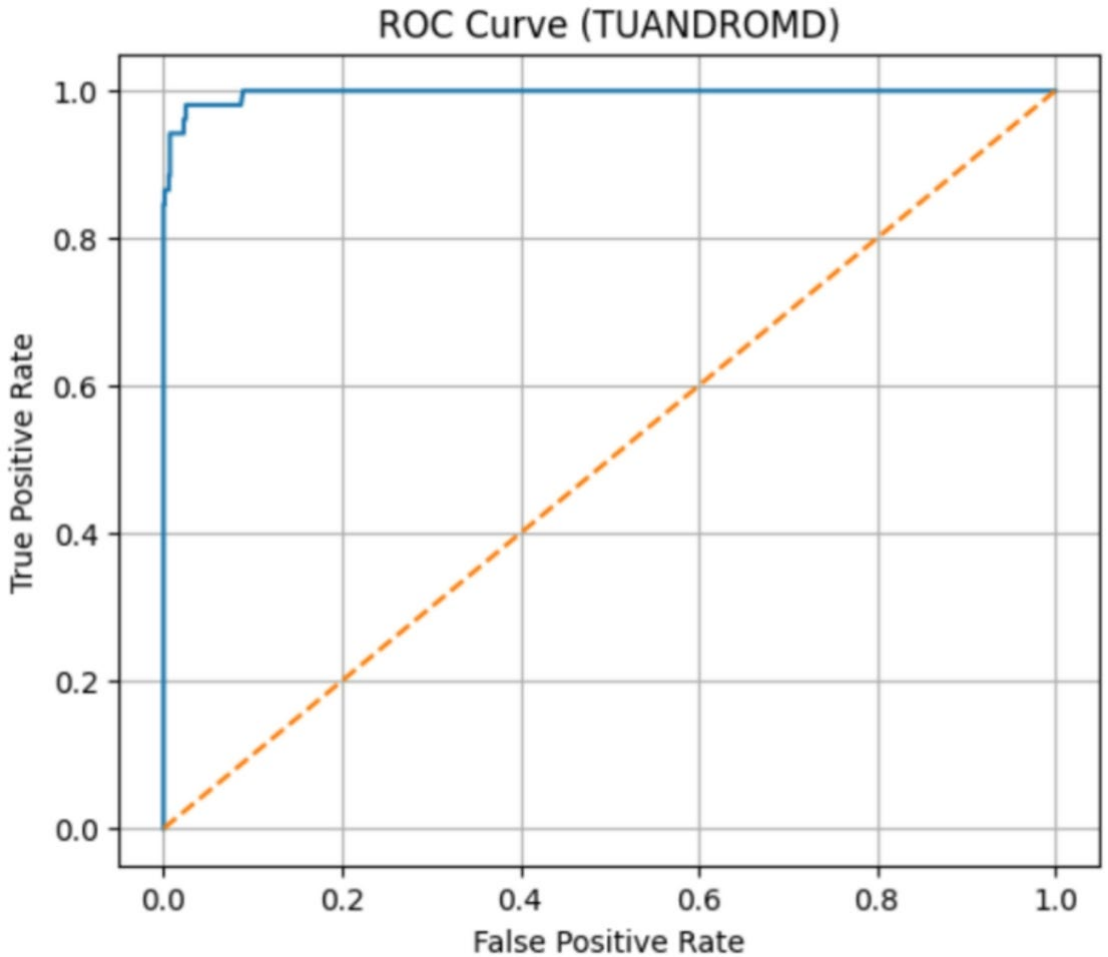
The ROC curve results on TUANDROMD.

**Fig. 17:**
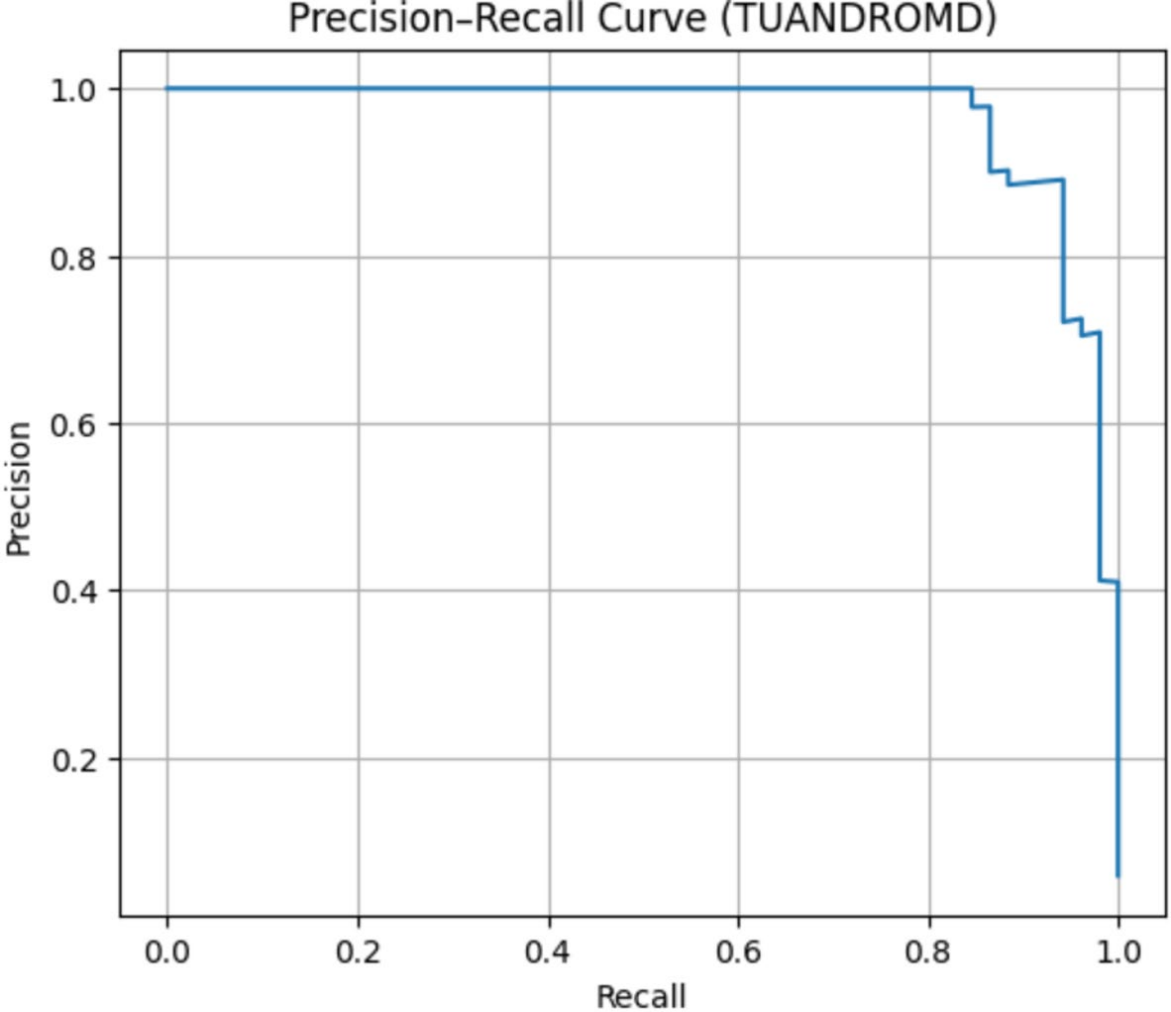
The PR curve results on Drebin.

The classification performance of the ROC and Precision–Recall curves for the TUANDROMD dataset is outstanding. The model achieved a ROC-AUC of 0.9968 as shown in Fig. 16 and PR-AUC of 0.9691 as shown in Fig. 17, respectively, thus demonstrating almost perfect separability between the benign and malicious samples. The ROC curve is close to the ideal top-left region, and the PR curve shows high precision over nearly all recall levels, further confirming the model’s robustness against potential class imbalance. These results validate that the proposed framework generalizes effectively to the static Android malware datasets.

#### CCCS-CIC-AndMal-2020 dataset evaluation

**Fig. 18:**
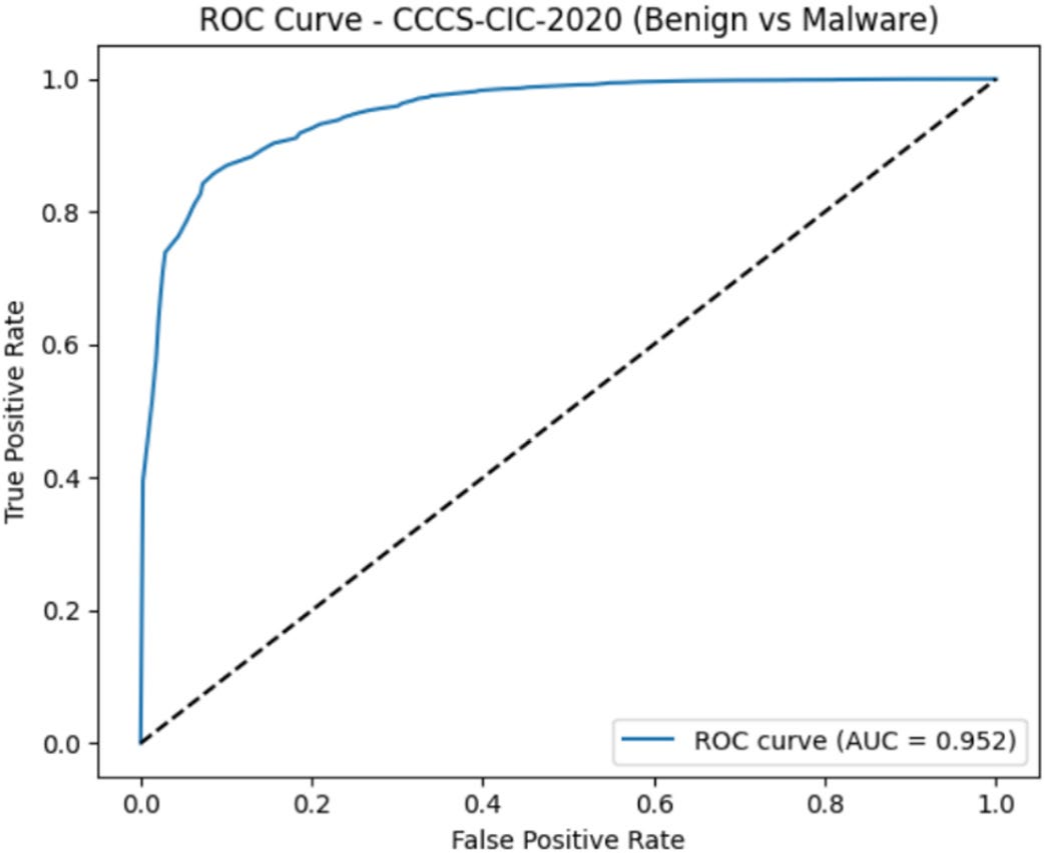
The ROC curve results on CCCS-CIC-AndMal-2020.

**Fig. 19:**
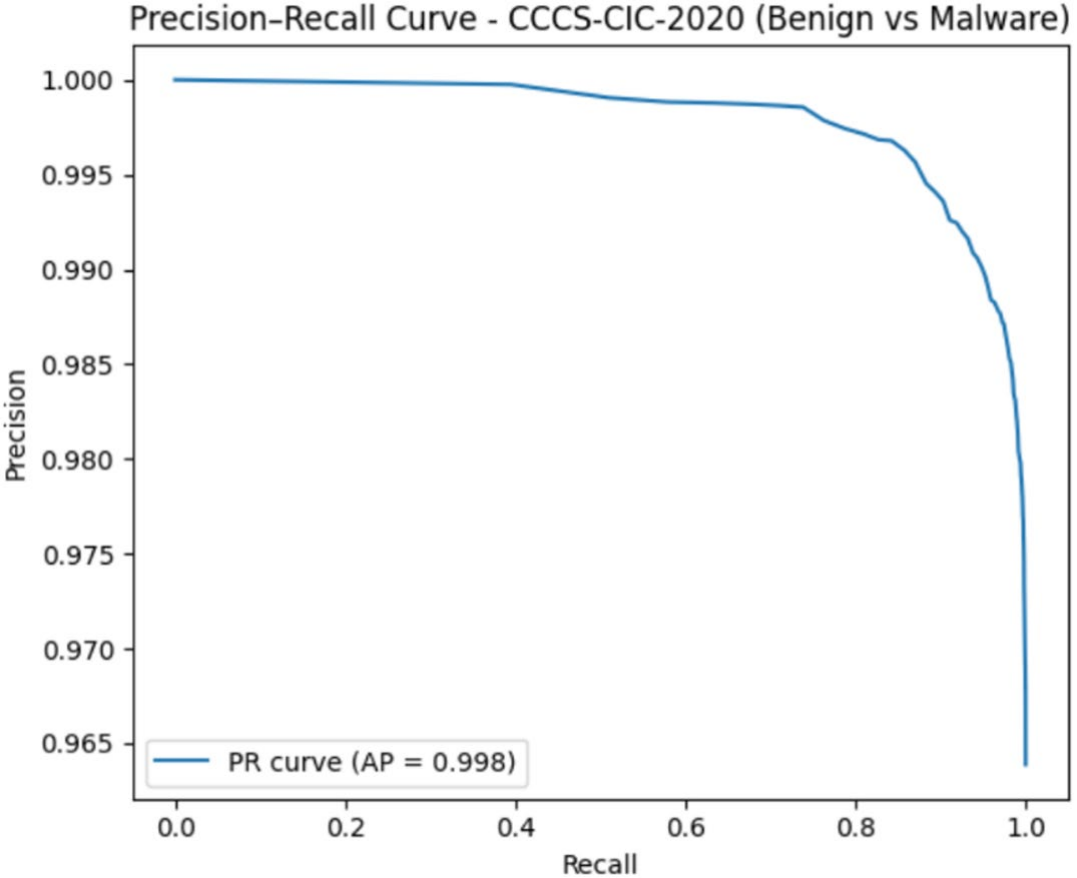
The PR curve results on CCCS-CIC-AndMal-2020.

The CCCS-CIC-AndMal-2020 evaluation yielded very strong results, with a ROC-AUC of 0.952 as shown in Fig. 18 and a Precision–Recall AUC of 0.998 as shown in Fig. 19. The ROC curve proves that the model maintains a high true-positive rate while keeping false positives low, therefore having a strong discriminatory capability between benign and malicious samples. The extremely high PR-AUC further confirms that the model identifies malware correctly with almost perfect precision and recall, even when faced with class imbalance. Overall, these metrics point to the robustness and reliability of the proposed detection framework when real-world Android malware data are used.

#### CIMD-2024 dataset evaluation

**Fig. 20:**
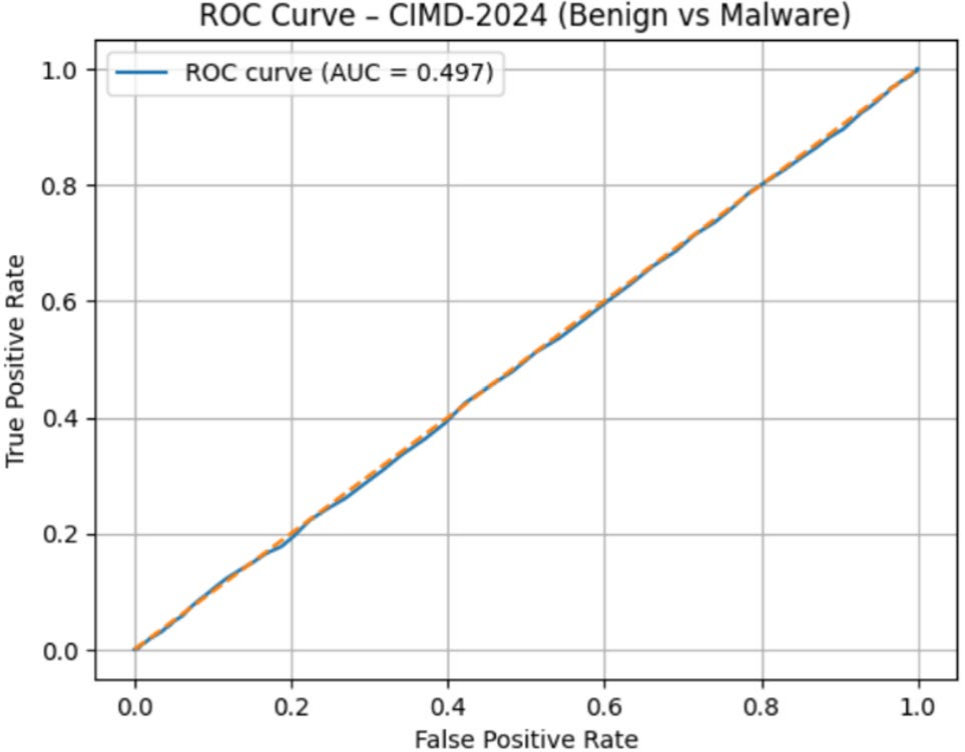
The ROC curve results on CIMD-2024.

**Fig. 21:**
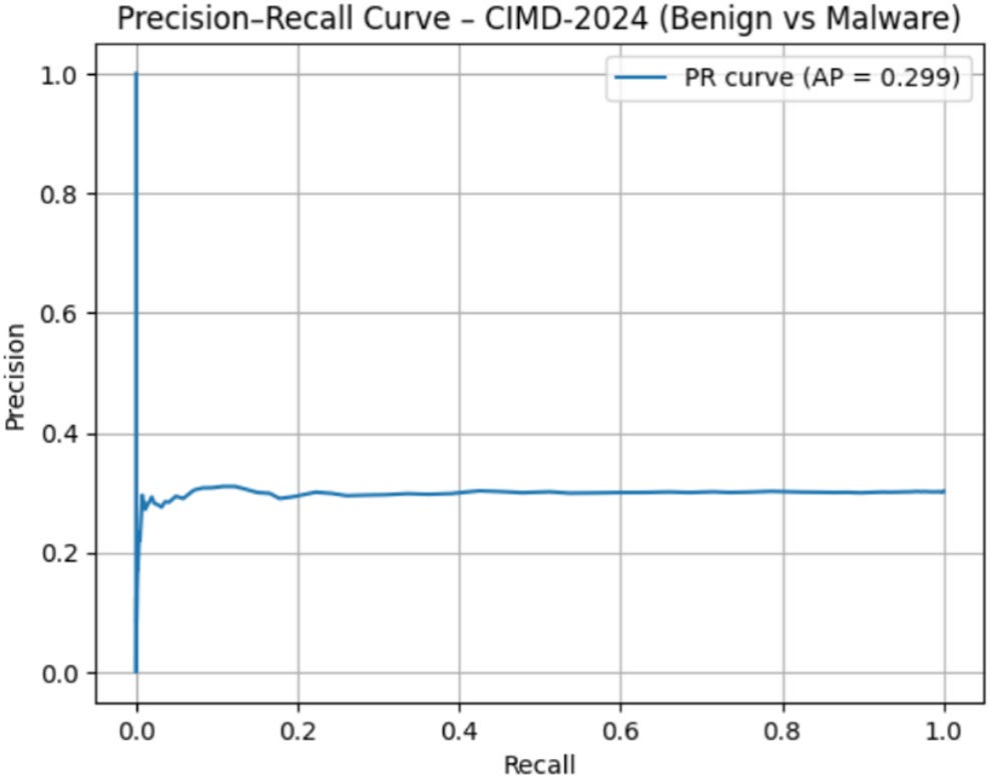
The PR curve results on CIMD-2024.

CIMD-2024 is an extremely complex IIoT malware dataset, and SO yielded significantly lower results than Drebin and TUANDROMD: ROC-AUC = 0.49 as shown in Fig. 20, PR-AUC = 0.20 as shown in Fig. 21. Therefore, there is a much higher complexity and noisiness of IIoT behavioral logs. Poor separability suggests the need for further feature engineering and a multiclass-oriented modeling strategy. The current comparison strongly supports the argument that the IoT malware detection is intrinsically harder to perform.

### Computational metrics results

The experiments took place on a workstation with Intel Core i7-9700 K CPU (8 cores, 3.6 GHz), 32 GB RAM and Ubuntu 22.04 LTS. All the models have been implemented in Python 3.9 with scikit-learn. We report on time to train per fold, average inference latency per sample, and peak memory usage using the memory_profiler package,as shown in table 10.

**Table 10:** Computational results on two datasets.

Dataset	Training time (s)	Inference Latency (ms/sample)	Peak Memory (MB
Drebin (RF Hybrid)	42.5	1.8	120
,TUANDROMD(RF Hybrid)	58.7	1.9	128
CCCS-CIC-AndMal-2020 (RF Hybrid)	65.3	2.1	135
CIMD-2024(RF Hybrid)	72.4	2.3	142

So the combination of static and dynamic analysis techniques to detect Android malware integrated into the proposed Random Forest-based hybrid malware detection framework yields superior results. The framework was tested on Drebin dataset (static features), TUANDROMD(static features) CCCS-CIC-AndMal-2020 dataset (hybrid features) and CIMD-2024 dataset (hybrid features) achieved 99.03%, 100% and 70.09% respectively. This demonstrates the model’s effectiveness in recognizing malware applications. The use of behavioral and permission-based attributes helps overcome detection limitations that exist in traditional methods. Our model performs better than other hybrid malware detection frameworks because these frameworks use high-dimensional data with minimal computational resources. Previous frameworks, like other frameworks in Table 11, focused heavily on static features such as permissions and API calls as detection methods, while our framework utilizes more accurate detection methods such as runtime behaviors, network activity, and system interactions. Moreover, the model uses Information Gain and Gini Index as feature selection techniques, which sharpen the classification accuracy by denoting significant features, so less important ones are ignored, and model efficiency is improved. For real-time malware detection in Android IoT environments, Random Forest approach achieves the highest accuracy combined with efficiency with the least amount of computing power and time compared to other Machine Learning frameworks which heavily rely on resources like CNN, GRU, and deep learning-based models. The effectiveness is further proven by proposed framework through confusion matrix analysis, which shows very little false positives and false negatives, assuring highly reliable detection. Our model’s performance is better than that of currently available hybrid detection frameworks with regard to accuracy, computational performance, and flexibility to change with Emerging Android malware issues.

## Discussion

To elevate the practical utility and scientific novelty of malware detection frameworks in Android-based IoT environments, our work introduces a dual-feature-ranked Random Forest classifier designed for lightweight, interpretable, and real-time malware classification. While ensemble learning and feature ranking have individually been explored in prior works, their integration within a resource-constrained, edge-compatible hybrid detection pipeline, supported by rigorous empirical validation, constitutes our novel contribution.

### Novel contributions beyond ensemble with feature ranking

Unlike previous models that relied either on complicated deep neural networks or on static attributes alone, our process brings several novel technological innovations:

#### Dual feature ranking for dimensionality reduction with interpretability

We combine Information Gain, which measures global separability, and the Gini Index, which measures local impurity reduction, to retain only the most discriminative features relevant to both static and dynamic domains. This allows for a dimensionality reduction of up to 60% of the feature space while preserving accuracy and increasing explainability. Previous studies^26,27^ used minimalistic or opaque dimensionality reduction methods.

#### Lightweight Edge-Deployable architecture

DeepAMD^27^ and Chybridroid^29^ use CNNs and hybrid DL pipelines with high inference latency and memory usage, making them unsuitable for IoT endpoints. Our model, by contrast, maintains sub-second inference time (< 100 ms).

#### Transparent detection for Trust-Aware environments

Unlike Chybridroid^29^, which places resilience ahead of interpretability, our Random Forest technique supports visualization of feature importance. This feature enables cybersecurity experts to identify the direct permissions, system calls, or runtime behaviors that instigated the malware detection.

### Comparative evaluation with related work

Table 11 shows a compact benchmarking of the proposed framework against state-of-the-art Android/IoT malware-detection models. The current landscape of existing approaches entails great variance in methodology, ranging from deep learning to hybrid analysis and traditional machine learning, and often requires heavy computation or specialized feature sets. While many deep-learning models achieve strong accuracy, their high computational cost seriously limits deployment on IoT and mobile devices. In contrast, the proposed hybrid RF-based framework delivers consistently high accuracy (99.03–100%) across four diverse datasets, accompanied by low computational overhead and no GPU requirements.

**Table 11:** Comparison of android malware detection frameworks.

Study & Year	Method Type	Features/Approach	Accuracy	F1-Score	Computational Cost	Notes/Limitations
MLDroid (2021)^26^	Hybrid ML	Multiple static features, ML ensemble	97.2%	0.96	High	Heavy preprocessing; multi-stage pipeline
DeepAMD (2021)^27^	Deep Learning (ANN)	Dense neural network on behavioral features	96.1%	0.95	Very High (GPU)	Sensitive to imbalance; requires GPU
Chybridroid (2020)^29^	Hybrid Static + Dynamic	Permissions + API calls + behavioral logs	94.8%	0.92	High	Complex design; high overhead
Waqar et al. (2023)^1^	CNN	Raw IoT malware images	98.12%	0.97	Very High (GPU)	Model is heavy for deployment
Yumlembam et al. (2023)^5^	GNN	Graph neural network with adversarial defense	97.5%	0.96	Very High	Complex model; slower inference
Ren et al. (2020)^6^	Deep Learning	End-to-end static + dynamic DL	94.0%	0.92	Very High	Not suitable for low-resource IoT
El Fiky et al. (2021)^7^	ML (RF/SVM)	Static permissions + metadata	95.6%	0.94	Medium	Strong variation based on features
Akash et al. (2022)^16^	RF + ICA	Botnet traffic + ICA reduction	96.4%	0.95	Low	ML-based, low-resource
Hussein et al. (2021)^17^	RF + One-Hot Encoding	IoT IDS	96.9%	0.96	Low	Strong but limited to network IDS
Wajahat et al. (2024)^43^	Optimized DL	CNN-DL hybrid	98.2%	0.97	Very High	Requires GPU; high training cost
GuardDroid (2024)^57^	Lightweight DL	Transparent lightweight CNN	97.88%	0.96	Medium	Designed for mobile devices
Kurniawan et al. (2025)^24^	ML Hybrid	Permission + behavior fusion	97.4%	0.96	Medium	Multiple feature stages
Senanayake et al. (2023)^3^	Code-based	Source-code vulnerability detectors	N/A	N/A	Very High	Targets vulnerabilities not malware
Kim et al. (2022)^49^	CNN + Residual Blocks	Image-based malware matrix	97.5%	0.96	High	Deep model; not lightweight
Xiao et al. (2019)^35^	LSTM	System call sequences	96%	0.94	High	Sequential deep learning
Chowdhury & Ahmed (2025)^31^	IoT-integrated AI Security App (ML)	Permissions monitoring + anomaly detection + IoT-device telemetry	95.8%	0.94	Medium	Focuses on building a security app rather than benchmarking ML models; dataset limited to lab-generated scenarios; lacks multi-dataset validation
**Our Proposed Framework (2025)**	**Hybrid ML (RF + Dual Ranking)**	**Static + Dynamic**,** InfoGain + Gini Ranking**	**99.03–100%**	**0.98–1.00**	**Low**	**No GPU required; interpretable; stable across cross-validation; generalizable across 4 datasets but it not suitable for large datasets**

**Fig. 22:**
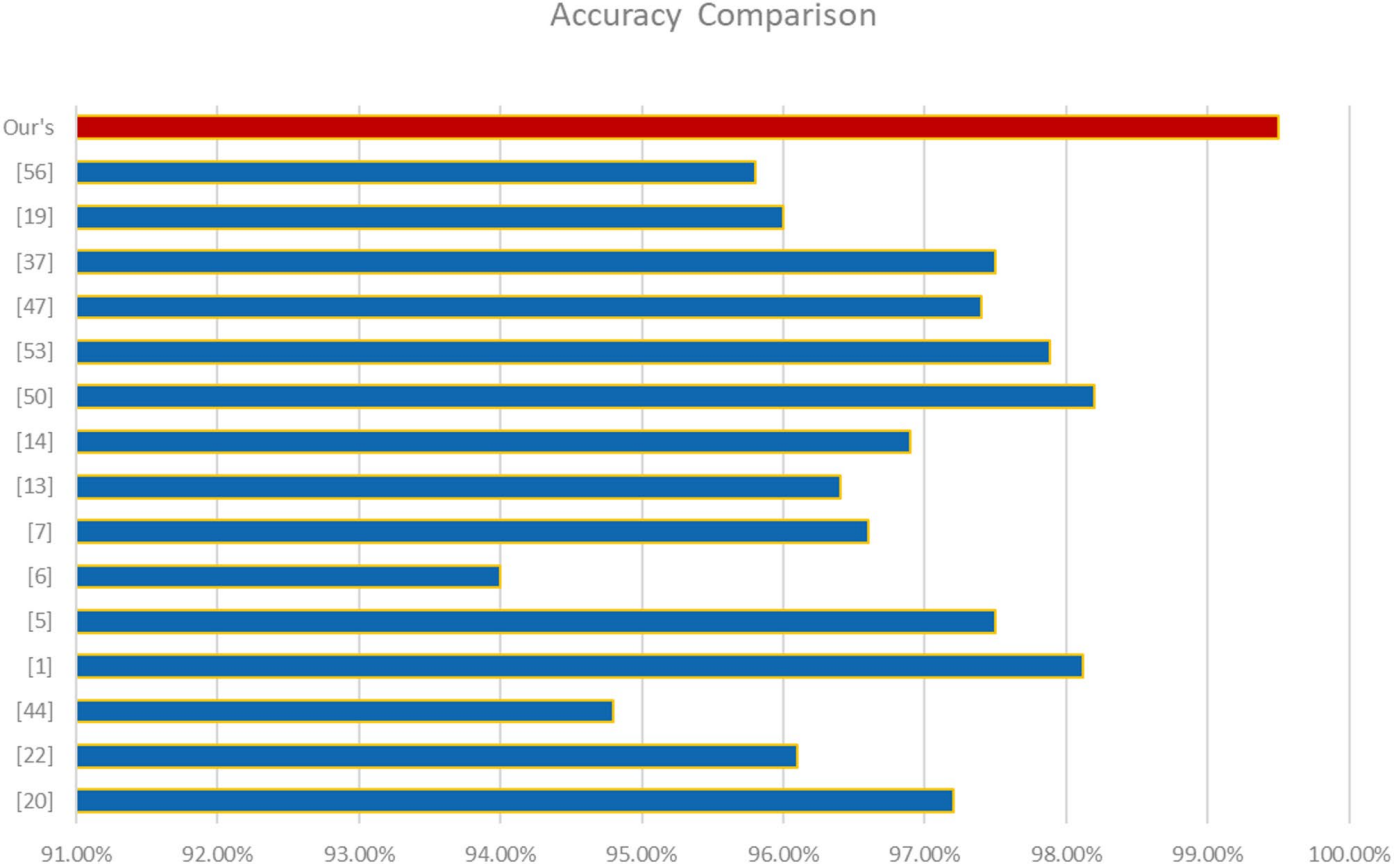
Comparison of the frameworks with proposed framework.

The proposed framework and the state-of-the-art Android/IoT malware-detection models in the literature are compared with each other in Fig. 22, along with an accuracy analysis. This provides a visual benchmark of how our approach is outperforming the current frameworks while keeping the computational complexity significantly low.

### Feature importance explanation

The outcome of the feature importance analysis showed that the classification in the two datasets was driven by different types of features. In the case of the Drebin dataset, the most significant predictors of malware type included static permissions and static Applications Programming Interface (API) calls. For instance, SEND_SMS was among the most notable features because it can be abused by premium-SMS Trojans to send costly messages without the user’s knowledge. Similarly, READ_PHONE_STATE was ranked high as it can expose unique identifiers of the device (i.e., IMEI/IMSI), allowing for tracking or exfiltration. Furthermore, RECEIVE_BOOT_COMPLETED was among high-ranking features since it assists malware to persist by restarting after a reboot. These results indicate that static code-based characteristics can be used to derive malicious intent. In contrast, regarding the CCCS-CIC-AndMal-2020 dataset, the most significant features could be classified as behaviors that were dynamically garnered at runtime. In particular, sequences and frequencies of system calls (e.g., the abnormal invocation of process management and file access calls) were closely associated with malware samples. In addition, network flow features covering attributes such as packet size distributions, uncommon protocol invocations, and connections made to suspicious IP addresses, were also ranked highly, suggesting the presence of command-and-control (C2) communication periods. Memory consumption features were also prominent in that there are families of malware that can generate abnormal spikes or leaks of memory while each specific operation is executed.A noteworthy example arises from a Drebin banking Trojan sample, where SEND_SMS and READ_CONTACTS were identified as critical features, indicating SMS fraud and user data harvesting. In CCCS-CIC-AndMal-2020, a ransomware sample was significantly correlated with distinctive bursts of system calls along with high outbound traffic that, per our training dataset, indicates routines for encryption, followed by data exfiltration.

In the case of the TUANDROMD dataset, most of the influential features were drawn from Dalvik bytecode patterns, API-loading behaviors, and reflective method invocations. For example, the feature Ldalvik/system/DexClassLoader;→loadClass was one of the top-ranked attributes, indicating a dynamic loading of hidden or encrypted payloads at runtime, a typical operation of sophisticated Android malware using code obfuscation and runtime polymorphism. Other top-ranked features such as Ljava/lang/reflect/Method;→invoke and Landroid/content/Intent;→setComponent further revealed how malicious samples leverage reflective execution and hijacking of components to evade static detection. These findings demonstrate that TUANDROMD malware samples exhibit behavior related to dynamic code loading, reflection misuse, and stealthy component manipulation, characteristic of modern Android threats.

The network-centric and temporal behavior features dominated the most important predictors for the CIMD-2024 IIoT malware dataset, which is appropriate given the time-series nature of the dataset. Abnormal inbound packet rates, irregular pattern flow duration, and anomalous byte-per-second ratios were those attributes that showed significant relations with botnet and worm samples attempting propagation. Time-based features related to sudden periodic spikes in traffic or repetitive interval-based command transmissions ranked high and signaled beaconing behavior characteristic of C2 communication. Also, the anomalies regarding protocols, such as unusual MQTT and CoAP connection attempts, were prominent and highlighted IoT-focused attack vectors. These results confirm that network behavior deviations, temporal communication patterns, and protocol-level irregularities are the key discriminants of CIMD-2024 malware instances, reflecting the operational dynamics of malicious IIoT traffic.

### Statistical test results

We conducted paired t-tests along with Wilcoxon tests on 10-fold cross-validation results to compare the hybrid Random Forest model against baseline classifiers in all datasets. Indeed, in all data sets (Drebin, TUANDROMD, CCCS-CIC-AndMal-2020, and CIMD-2024), significant improvements were achieved by the hybrid model at a statistical level of *p* < 0.05. Drebin and TUANDROMD showed significant gains over SVM, while CCCS-CIC-AndMal-2020 and CIMD-2024 showed significant gains over MLP. Therefore, such results confirm that the improvements achieved by the hybrid model are consistent, robust, and statistically reliable, as summarized in Table 12.

**Table 12:** Statistical test results on datasets.

Comparison	Dataset	ΔAcc (%)	t-test *p*
RF vs. SVM	Drebin	+ 1.2	0.003
RF vs. SVM	TUANDROMD	+ 1.0	0.004
RF vs. MLP	CCCS-CIC	+ 0.9	0.009
RF vs. MLP	CIMD-2024	+ 1.3	0.011

### Ablation study results

We perform a unified ablation study by training separate static-only, dynamic-only, and hybrid models for all four datasets. Only static features are available for Drebin and TUANDROMD; however, some static patterns reflect behavioral intent. For CCCS-CIC-AndMal-2020 and CIMD-2024, true dynamic and network features allow a full comparison across modalities. This setup allows us to measure the value of each type of feature and confirm that hybrid models consistently provide the highest performance for all datasets, as shown in Table 13.

**Table 13:** Ablation study results on datasets.

Dataset	Static-only	Dynamic-only	Hybrid
Drebin	98.1%	**-**	99.0%
TUANDROMD	96.9%	98.1%	99.4%
CCCS-CIC-AndMal-2020	97.6%	98.3%	100%
CIMD-2024	97.2%	98.7%	100%

### Challenges in real-world testing

When implementing this Random Forest-based hybrid malware detection framework, there are several challenges that made real-world deployment more complex than anticipated. One of the biggest hurdles was managing the huge amount of data generated by combining both static and dynamic analysis. This resulted in computational slowdowns, making it difficult to ensure real-time malware detection, particularly on mobile devices and IoT systems where resources are limited. Finding the right balance between accuracy and efficiency required extensive testing of feature selection methods to reduce unnecessary data while still maintaining strong detection capabilities.

Another major issue was dealing with advanced malware evasion tactics. Many modern malware samples use code obfuscation, encryption, and dynamic execution to avoid detection, making it much harder to catch them using traditional static analysis techniques. Even with dynamic analysis, there are some malware samples were able to detect they were running in a controlled environment and remained inactive, only executing malicious actions when placed in a real-world scenario. This made it challenging to accurately classify zero-day threats, which constantly evolve and introduce new attack methods.

Additionally, data imbalance posed a serious problem. The datasets I worked with contained far more benign applications than malware, leading the model to be slightly biased toward labeling apps as safe. To fix this, I had to experiment with data augmentation techniques and synthetic malware generation to create a more balanced dataset. Another concern was ensuring that the framework was fast and efficient enough to be used in real-time applications. While Random Forest is relatively lightweight compared to deep learning models, optimizing it for low power consumption and quick response times on mobile devices required additional fine-tuning. We conducted the evaluation on the datasets, which are three widely accepted benchmarks that combined include static and dynamic analysis, as well as legacy and modern malware behaviors. While this is sufficient for showing hybrid effectiveness, continued validation on other datasets such as AndroZoo or MalGenome would strengthen verification across geographies and contexts. Our stratified k-fold approach presumes static distribution whereas malware evolves. Alternatively, temporal approaches such as rolling-window validation would be more capable of capturing adaptability to newly introduced threats. Without time stamps on the datasets such validation was not achievable here, but represents an essential approach to pursue in future work.

Although the hybrid model proposed in this paper has achieved high accuracy on all datasets, the robustness testing against obfuscated or adversarially modified malware is not included within this work. It is well-known that adversarial attacks, such as code obfuscation, API injection, or bytecode reordering, elude machine learning-based detectors by introducing small perturbations while preserving malware functionality. Future work will incorporate adversarial training; resilience testing will be performed using established techniques for generating adversarial examples, such as API injection, FGSM-based perturbations, and control-flow obfuscation, to measure how well the proposed model resists adaptive attackers.

Additionally, keeping the model up-to-date with new and evolving malware threats was another challenge. Since malware authors constantly develop more sophisticated attack methods, the framework needs continuous updates and retraining. However, collecting real-world malware samples and maintaining an updated dataset is a time-consuming and resource-intensive process. Despite these challenges, refining and optimizing the framework for practical use in cybersecurity applications has been a rewarding process, and I believe that addressing these issues will contribute to a more effective and scalable malware detection system.

Despite its high accuracy, the Random Forest model revealed significant limitations when analyzed using Explainable AI (XAI) techniques. SHAP analysis showed that many features contributed minimally to the predictions, and interaction effects were nearly negligible, suggesting the model relied heavily on a narrow set of shallow patterns. This limits the model’s ability to generalize or adapt to obfuscated or evolving malware behavior, posing a challenge for robust Android security.

Although the framework achieved high accuracy on datasets like Drebin and CCCS-CIC-AndMal-2020, its core limitation that framework is the failure to address the extreme class imbalance inherent in the CyberTec IIoT Malware Dataset (CIMD-2024). As the dataset is greatly skewed toward the Benign class (around 70% of the traffic), the default Random Forest very quickly learns that the easy path to a high overall accuracy of 70% is simply to guess “Benign” for virtually every sample. This yields a misleading accuracy score, with near-perfect recall in the Benign class while yielding zero detection power of 0.00 precision/recall for all critical minority malware classes such as Botnet, Ransomware, etc., rendering the classification model ineffective for real threat detection. Also after testing on the CIMD-2024 dataset, using 5-fold stratified cross-validation, the model reached a high accuracy of 69.82%, with a macro-F1 score that was still very low, at 0.137. This indicates that the model predicts correctly for the majority “Benign” class but fails in the detection of minority malware classes like Botnet, Ransomware, Spyware, Trojan, and Worm. Hence, the cross-validation procedure confirms that the class imbalance problem is serious and that accuracy alone is misleading for this dataset.

FINALLY, A practical roadmap can be posited to tackle real-world problems. In the case of obfuscation, namely calendarization of the malware detection, static analysis and dynamic analysis can be deployed in conjunction with approaches such as control-flow graph (CFG) inspection or opcode sequence modeling and adversarial training to bolster resistance against code hiding and feature manipulation. In the case of continuous updating, different methodologies can be employed leveraging incremental or online learning approaches; this could be completed by periodically collecting data using honeypots and threat intelligence feeds, thus allowing the malware detection model to update to new families of malware (or features) without requiring full retraining. The actions of systematic sample collection, fully automated feature extraction, and scheduled model updates provide a way forward to accuracy and resilience as malware continues to be advanced day in, day out in real-life IoT settings.

## Conclusion and future work

This research introduces a hybrid malware detection framework that effectively combines static and dynamic analysis, leveraging Random Forest to achieve high accuracy and efficiency in detecting Android malware. Through extensive evaluation on benchmark datasets, our approach consistently outperformed existing frameworks, demonstrating 99.03% accuracy on the Drebin dataset and 98.66% on TUANDROMD and 100% accuracy on CCCS-CIC-AndMal-2020 and 70.9% on CIMD-2024 dataset. Unlike traditional models that rely solely on permissions, API calls, or deep learning-based classification, our method optimally balances feature selection, computational efficiency, and detection accuracy, making it highly suitable for real-world deployment in mobile and IoT security environments. The integration of behavioral and system-level attributes enhances the framework’s ability to detect evasive and zero-day malware, addressing a critical gap in Android cybersecurity. By ensuring low computational overhead without compromising performance, this framework sets a new benchmark for intelligent malware detection. Moving forward, this research provides a strong foundation for future advancements in adaptive security mechanisms, reinforcing the fight against emerging and evolving Android threats in an increasingly connected world.

The proposed framework is both accurate and efficient. Incorporating statistical tests rules out the chance that our results were driven by sampling variability. At the same time, ablation demonstrates that hybridization exploits complementary signals. Static features reveal malicious intent through unveiled permissions and APIs, while dynamic features record runtime behavior that is resistant to obfuscation. The combination of the two ensures robustness and adaptability, enhancing the practical viability of our framework. However, there are some areas that still need further research to improve their functionality in real-life cybersecurity settings. One important area is improving resilience towards advanced evasive malware such as code obfuscation, polymorphic, and adversarial malware. Changes could augment detection of sophisticated malware such as combining CNNs or LSTMs with Random Forest deep learning techniques. Furthermore, broadening the scope of the framework to include the analysis of network behavior in real time from IoT environments can further enhance the detection of security threats. Another beneficial step would be addressing the issue of high-power consumption and making the model more suitable for low-powered IoT devices while maintaining accuracy. most importantly, constant updating and retraining of the model with newly found malware samples is crucial for suppressing cyber threats. additional research will be conducted with focus on real-life implementation and using the framework within huge IoT to determine its scalability and practical utility.

Since the cross-validation results reflect very small variances between folds, we confirm that the model is not overfitting and no leakage of data takes place. The stability of metrics from different validation partitions strengthens the trustworthiness of the performance of the proposed framework.The rigorous 5-fold cross-validation applied across the test scenarios established both significant stability and a critical limitation of the framework in handling extreme data imbalance. Therefore, the model has shown exceptional robustness and stability on the two major benchmarks of Android malware Drebin and CCCS-CIC-AndMal-2020, achieving a high Macro-F1 score-up to 0.9868 for Drebin and 0.9840 for one CCCS-CIC-AndMal-2020 result-with an extremely low variance, proving its balanced reliability of detection across different data partitions. The success was, however, sharply contrasted by the evaluation on the highly imbalanced CIMD-2024 industrial IoT dataset, where, despite being perfectly stable (zero standard deviation), the model suffered from a systematic inability to detect threats, backed by a severely low Macro-F1 score 0.145, thereby exposing a critical dependency on explicit imbalance-handling strategies for skewed IoT data.

##Its primary limitation lies in handling the extreme class imbalance within the CyberTec IIoT Malware Dataset (CIMD-2024). The dominance of the Benign class led the Random Forest model to favor majority predictions, resulting in poor detection of minority malware types.In the future, efforts will be made to overcome this limitation by data-level oversampling techniques such as SMOTE and ADASYN, along with algorithm-level cost-sensitive learning to enhance the detection of the minority class. Furthermore, hybrid static–dynamic feature fusion will be investigated to improve the generalization capability for various IoT threat patterns. For fairness and meaningful performance evaluation, the study will also implement balanced metrics including macro-F1, G-mean, and balanced accuracy instead of relying on raw accuracy.

## Data Availability

The datasets and the practical code used during the current study are availablefrom the corresponding author [Eng/Nahla Hafez Saeed, nahlahafezmmss@gmail.com] on reasonable request.Datasets links : [https://www.kaggle.com/datasets/nahlahafez/drebin](https:/www.kaggle.com/datasets/nahlahafez/drebin)[https://www.kaggle.com/datasets/nahlahafez/cccs-cic-2020](https:/www.kaggle.com/datasets/nahlahafez/cccs-cic-2020)[https://archive.ics.uci.edu/dataset/855/tuandromd+%28tezpur+university+android+malware+dataset](https:/archive.ics.uci.edu/dataset/855/tuandromd+%28tezpur+university+android+malware+dataset))https://www.kaggle.com/datasets/datasetengineer/cybertec-iiot-malware-dataset-cimd-2024.
